# Experimental Design to Evaluate Directed Adaptive Mutation in Mammalian Cells

**DOI:** 10.2196/resprot.3860

**Published:** 2014-12-09

**Authors:** Michael Bordonaro, Christopher R Chiaro, Tobias May

**Affiliations:** ^1^Department of Basic SciencesThe Commonwealth Medical CollegeScranton, PAUnited States; ^2^MetabolomicsCenter for Molecular Toxicology and CarcinogenesisThe Pennsylvania State UniversityUniversity Park, PAUnited States; ^3^Helmholtz Centre for Infection Research, Inhoffenstrasse 7, 38124BraunschweigGermany; ^4^InSCREENeX GmbH, Inhoffenstrasse 7, 38124BraunschweigGermany

**Keywords:** quantum, adaptive mutation, mouse embryo fibroblast

## Abstract

**Background:**

We describe the experimental design for a methodological approach to determine whether directed adaptive mutation occurs in mammalian cells. Identification of directed adaptive mutation would have profound practical significance for a wide variety of biomedical problems, including disease development and resistance to treatment. In adaptive mutation, the genetic or epigenetic change is not random; instead, the presence and type of selection influences the frequency and character of the mutation event. Adaptive mutation can contribute to the evolution of microbial pathogenesis, cancer, and drug resistance, and may become a focus of novel therapeutic interventions.

**Objective:**

Our experimental approach was designed to distinguish between 3 types of mutation: (1) random mutations that are independent of selective pressure, (2) undirected adaptive mutations that arise when selective pressure induces a general increase in the mutation rate, and (3) directed adaptive mutations that arise when selective pressure induces targeted mutations that specifically influence the adaptive response. The purpose of this report is to introduce an experimental design and describe limited pilot experiment data (not to describe a complete set of experiments); hence, it is an early report.

**Methods:**

An experimental design based on immortalization of mouse embryonic fibroblast cells is presented that links clonal cell growth to reversal of an inactivating polyadenylation site mutation. Thus, cells exhibit growth only in the presence of both the countermutation and an inducing agent (doxycycline). The type and frequency of mutation in the presence or absence of doxycycline will be evaluated. Additional experimental approaches would determine whether the cells exhibit a generalized increase in mutation rate and/or whether the cells show altered expression of error-prone DNA polymerases or of mismatch repair proteins.

**Results:**

We performed the initial stages of characterizing our system and have limited preliminary data from several pilot experiments. Cell growth and DNA sequence data indicate that we have identified a cell clone that exhibits several suitable characteristics, although further study is required to identify a more optimal cell clone.

**Conclusions:**

The experimental approach is based on a quantum biological model of basis-dependent selection describing a novel mechanism of adaptive mutation. This project is currently inactive due to lack of funding. However, consistent with the objective of early reports, we describe a proposed study that has not produced publishable results, but is worthy of report because of the hypothesis, experimental design, and protocols. We outline the project’s rationale and experimental design, with its strengths and weaknesses, to stimulate discussion and analysis, and lay the foundation for future studies in this field.

## Introduction

### Adaptive Mutation

Random biological mutations occur independent of selection pressure. Although this likely describes most mutations, adaptive mutation may also contribute to genetic variability in changing environments. In adaptive mutation, the genetic change does not exist independent of the selective pressure; instead, the presence and type of selection influences the frequency and character of the mutation event. Evidence for adaptive mutation exists for both bacteria and yeast, and possibly for prostate cancer cells; researchers believe that adaptive mutation contributes to the evolution of microbial pathogenesis, cancer, and drug resistance, and may become a focus of novel therapeutic interventions [1-22].

This proposal evaluates the possibility of directed adaptive mutation in mammalian cells. Our objective is to distinguish between 3 types of mutation: (1) random mutations independent of selective pressure; (2) undirected adaptive mutations, which arise when selective pressure induces a general increase in the mutation rate; and (3) directed adaptive mutations, which arise when selective pressure induces targeted mutations that specifically influence the adaptive response.

A number of hypotheses have been postulated to explain undirected adaptive mutation. These include replication and recombination systems, slow repair of mismatched bases, mutagenic transcription, and gene amplification/duplication (reviewed in [17]). The most cited potential mechanism for undirected adaptive mutation is induction of a transient hypermutagenic “mutator phenotype,” in which the mutation frequency is increased by up to several orders of magnitude [4,6,18]. The mutator phenotype has been invoked to explain the development of resistance to the androgen receptor antagonist bicalutamide in prostate cancer cells [16], a possible example of undirected adaptive mutation. LNCap prostate cancer cells respond to a bicalutamide challenge by upregulating expression of error-prone DNA polymerases and downregulating expression of high-fidelity DNA polymerases and mismatch repair (MMR) proteins, resulting in an increased mutation rate [16]. However, we are interested in evaluating the possibility of directed adaptive mutation, defined as matched specific mutations with associated specific environmental changes (eg, the targeted mutation of one gene to a single specific selective pressure). This hypothesized form of adaptive mutation cannot be explained through a generalized increase in mutation rate.

### Basis-Dependent Selection and Quantum Biology

Ogryzko [23-26] and, more recently, Bordonaro and Ogryzko [27] proposed a quantum biological mechanism for adaptive mutation (Multimedia Appendix 1) in which superposition is context-dependent. In biological systems, context-dependence describes possible environmental conditions; thus, a change of environment represents a change in the basis (ie, basis transformation) describing a quantum mechanical system. Whether or not a quantum state is in a state of superposition (quantum coherence) is strictly dependent on the basis used to describe the system. A state that can be described as being in superposition in one basis may not be so described in another basis, and vice versa*.* Thus, the state of a biological system can be stable in a given environment and not affected by decoherence; however, upon a change in environment (change of basis), that same biological system can be described as being in a state of superposition. Decoherence would follow, resulting in a new set of stable, preferred states, one of which would be observed on measurement. Because there is no requirement to maintain quantum coherence for some arbitrary time period in any arbitrary environment, past criticisms [28] of quantum biology (eg, “biological systems are too warm and complex to maintain quantum coherence”) are not relevant.

Quantum superposition is known to contribute to several biological processes (reviewed in [27]); however, the role of quantum effects in adaptive mutation has not been established. The hypothesis of basis-dependent selection attempts to address this deficiency through a novel model of directed adaptive mutation [27]. Thus, consider 2 cell states (A_1_ and A_2_, with A_2_ representing a cell state characterized by a gene mutation allowing for cell growth) and consider 2 possible environmental states (B_1_ and B_2_, such that only B_2_ allows for cell growth). Cell growth will occur only with cell state A_2_ in environment B_2_. A mutation that enables cell growth (A_2_) would occur only (1) in an environment suitable for cell growth (B_2_) and (2) only after exposure to that specific environment. Therefore, each specific microenvironment is correlated with a specific set of potential cell states (eg, cell states characterized by wild-type or mutant DNA sequences). These mutations do not occur randomly; instead, the cellular microenvironment (B_2_) selects the possible spectrum of cell states (A_1_ and A_2_) possible in that environment. An irreversible change in the state of the cell (eg, clonal expansion) would establish state A_2_ as that which is observed. If A_2_ represents a mutant cell state, this process can be described as directed adaptive mutation A_2_ induced by the selective pressure of environment B_2_. Note that the selection process is dependent on the environment of the biological system; hence, it is basis-dependent selection leading to adaptive mutation.

It is acknowledged that adaptive mutation, including what we define here as “directed” adaptive mutation, could in each case be dealt with through theories invoking classical mechanisms, particularly when ad hoc explanations are used. However, we believe that the difference between progressive and regressive research programs, previously discussed with respect to basis-dependent selection [27], is of relevance. The hypothesis of basis-dependent selection makes testable predictions, which would be evaluated via our experimental design. Specific findings (eg, directed adaptive mutation in our system) would be better explained by quantum than by classical mechanisms, and the basis-dependent selection hypothesis leads to further testable predictions that can be evaluated by experiments extending our approach. Future refinements in methodology may more definitively identify the fundamental nature (eg, quantum vs classical) of the mechanisms driving adaptive mutation. This would test predictions generated by the findings of the study outlined here, leading to more refined hypotheses and additional testable predictions. There is inherent value in proposing, and testing to the extent we are able, basis-dependent selection as a mechanism driving adaptive mutation. Thus, our experimental design is informed by the progressive research program suggested by previous discussions of basis-dependent selection [23-27].

### Experimental Design

In our system, expression of SV40 large T antigen (TAg) is required for clonal cell growth, and expression of TAg is dependent on environmental conditions. Thus, we use an inducible system in which TAg expression and resultant clonal cell growth occurs in the presence of doxycycline. However, the TAg expression cassette is designed to contain a mutation that prevents TAg expression; therefore, unless the mutation is reversed, clonal cell growth would not occur even in the presence of doxycycline. Thus, in this system, clonal cell growth requires (1) doxycycline and (2) that the inactivating mutation is reversed by a countermutation allowing TAg expression. In the absence of doxycycline, the cells would not grow regardless of countermutation; conversely, in the absence of countermutation, treatment with doxycycline would not induce cell growth. Therefore, our prediction is that countermutation (ie, another mutation) would occur in a directed manner dependent on the appropriate environmental conditions for growth (ie, doxycycline).

This experimental model can be theoretically interpreted according to basis-dependent selection [27] as follows. In environment B_1_ (no doxycycline), cell states A_1_ (no countermutation) and A_2_ (countermutation) cannot be distinguished because cell growth cannot occur in the absence of doxycycline. However, in environment B_2_ (presence of doxycycline), the 2 cell states can be distinguished because only cell state A_2_ is capable of responding to doxycycline with clonal growth. In the doxycycline-treatment environment, the 2 “A” states, which can now be distinguished, are stable, preferred states of the cell system. Therefore, in the presence of doxycycline, the superposition of cell states A_1_/A_2_ evolves through (1) decoherence into the 2 stable, preferred states, followed by (2) observation of clonal growth (cell state exhibiting countermutation A_2_) through the irreversible process of proliferation of cells exhibiting state A_2_.

**Figure 1:**
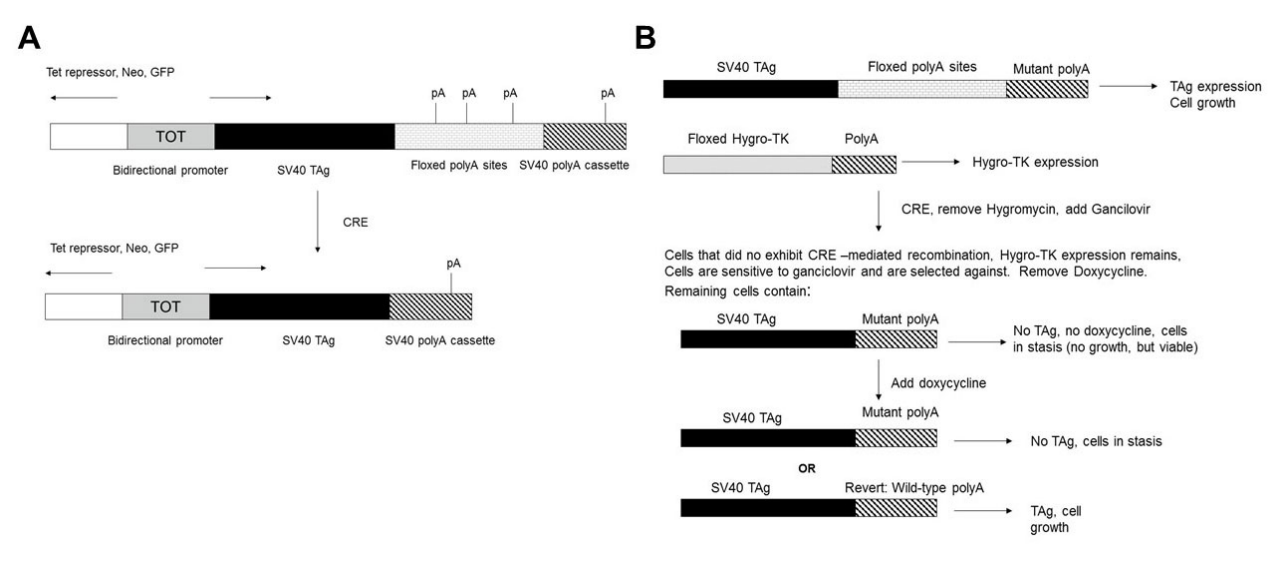
Basic strategy for pRITAGKHexa experiments. (A) Plasmid structure. (B) Outline of the experimental approach.

Using this approach, we have devised an experimental system based on inducible and reversible cell immortalization. In this setting, mouse embryo fibroblast (MEF) cells require TAg expression for immortalization and clonal growth. MEF cells will be stably transfected with a TAg expression plasmid (Figure 1) that allows for clonal growth dependent on doxycycline-induced TAg expression. In the absence of doxycycline, cells should not express TAg or undergo clonal growth, while maintaining the ability to exhibit efficient growth (“reimmortalization”) if doxycycline is added to the growth medium [29]. The structure of the plasmid is shown in Figure 1. A bidirectional promoter allows for expression of tetracycline (Tet) repressor, neomycin-resistance gene, and green fluorescent protein (GFP) in one direction, and for expression of TAg, in a doxycycline-inducible manner, in the other direction [29]. Because these MEF cells require TAg for continuous growth, the cells must express TAg for routine cell culture and cell expansion. Therefore, a characteristic of our system is that (1) wild-type TAg expression can be used to expand the cells as needed, but (2) the inactivating mutation is introduced at the start of the experimental protocol, eliminating TAg expression when desired. The mutation utilized is in the polyadenylation (polyA) site hexanucleotide sequence for the TAg expression cassette.

**Figure 2:**
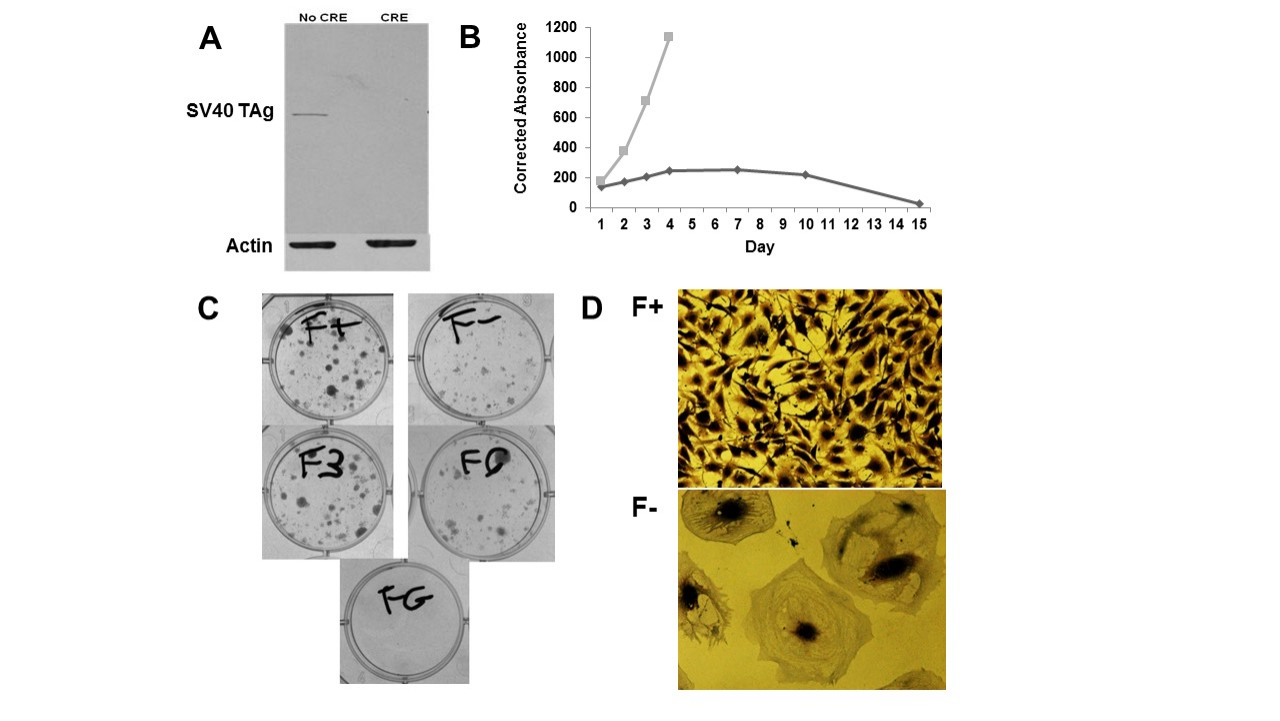
Preliminary data on system characteristics. (A) Repression of TAg expression after CRE-Lox excision of wild-type polyA cassette. (B) Doxycycline-dependent cell proliferation. Gray squares show induction; black diamonds are controls. (C) Doxycycline-dependent clonal cell growth and reanimation. Induced (F+) and not induced (F–) cells are shown. For samples F3 and F6, cells were incubated without doxycycline for 3 or 6 days, respectively, and then doxycycline was added to the media. (D) Typical cellular phenotypes from samples F+ (with doxycycline) and F– (no doxycycline) are shown.

**Figure 3:**
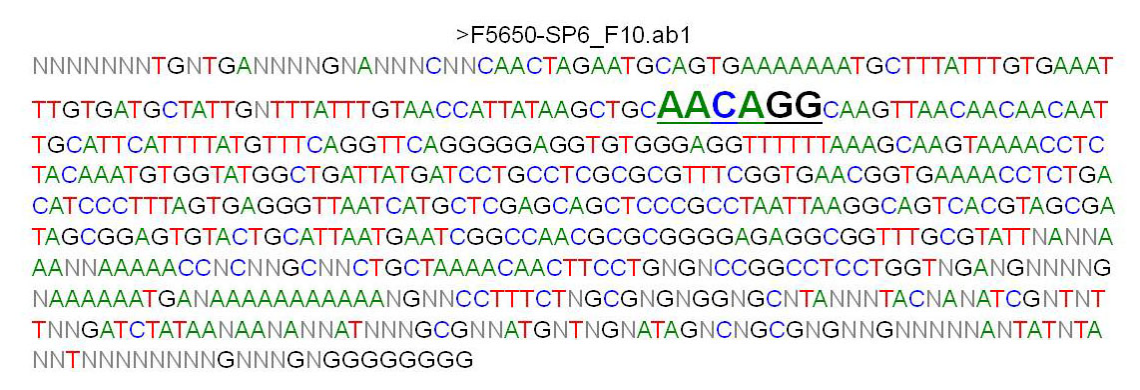
Sequencing data for clone F. Sequencing of F clone target polyA site; PCR product sequencing from genomic DNA. Relevant AACAGG sequence is in larger, bold font, and underlined.

The controlled pattern of TAg expression will be accomplished by inserting tandem polyA sites downstream of the TAg sequence (Figure 1). Immediately downstream of TAg is a floxed (“flanked by lox sites”) polyA cassette containing 3 wild-type SV40 (L) polyA sites. This functional polyA cassette allows for TAg expression and cell growth during routine cell culture before the start of the experiment. Further downstream is another SV40 (L) polyA site containing a mutated hexanucleotide sequence. The polyA site mutation is designed to repress 3′ end processing [30], eliminating TAg expression, and inducing cell quiescence at the start of the experiment. When the upstream wild-type polyA cassette is present, the transcripts undergo efficient 3′ end processing, allowing for TAg expression and MEF cell growth (Figure 2). However, when the wild-type polyA cassette is removed by CRE-Lox excision, only the mutated inactive polyA site (Figures 1-3) remains. The resulting defect in 3′ end processing should repress TAg expression and inhibit MEF cell growth. Clonal cell growth would occur only on reversal of the polyA site mutation, restoring efficient 3′ end processing and TAg expression in the presence of doxycycline.

Therefore, an outline of the overall experimental design for this project (Figure 1) is as follows. pRITAGKHexa and a plasmid containing a floxed hygromycin-thymidine kinase (Hygro-TK) expression cassette would be stably cotransfected into MEF cells and the cells incubated with G418, hygromycin, and doxycycline. TAg expression allows for cell growth. Removal of the floxed SV40 polyA sites from the integrated RITAGKHexa sequences, and the Hygro-TK cassette from the integrated Hygro-TK plasmid, would be achieved by CRE expression from an adenovirus (Figure 1). Thus, when cells transfected with the experimental plasmid are exposed to CRE recombinase, the floxed polyA sites are removed, leaving behind a mutant SV40 polyA site as the only polyA site for TAg expression. Cells would be taken off hygromycin and subsequently treated with ganciclovir to kill cells that did not undergo CRE-mediated excision (and still express TK). At the same time, doxycycline is removed from the media and the cells enter a potentially reversible stasis. Doxycycline would then be added back to the media; cells that retain the mutant polyA site would not efficiently express SV40 TAg and would not grow, but those cells that exhibit a further “reversion” mutation to wild-type polyA sequence would reexpress SV40 TAg and commence growth. If the latter occurs, follow-up experiments would determine whether the mutation to wild-type polyA status was adaptive or preexisting.

Thus, the initial characterization data derived from pilot experiments clearly show that the basics of the system are working as expected, for SV40 TAg expression and cell growth (Figure 2), as well as for the presence of the correct mutant polyA sequence in the stably transfected MEF cells (Figure 3). The experimental protocols utilized to generate these preliminary data were as follows. For analysis of SV40 TAg protein expression, MEF cells stably transfected with the pRITAGKHexa and Hygro-TK plasmids were infected or mock infected with Ad-CRE adenovirus at a multiplicity of infection (MOI) of 1000. Cells exposed to Ad-CRE were treated with 50 μM ganciclovir to eliminate cells that were not infected and did not undergo CRE-Lox excision. Three days later, protein was isolated and Western blot analysis performed with anti-SV40 TAg and anti-actin antibodies. SV40 TAg is expressed before (no CRE), but not after (CRE), excision of the wild-type polyA cassette; excision leaves behind only the mutant hexanucleotide polyA site. To analyze doxycycline-dependent cell proliferation, MEF cells were plated out at 4000 cells/well in a 96-well plate either in the presence of 4 μg/mL doxycycline, 400 μg/mL G418, and 100 μg/mL hygromycin (+Doxy) or in the presence of hygromycin alone (–Doxy). Note that the neomycin-resistance gene expression is also under the control of the doxycycline-inducible promoter, so the –Doxy samples are incubated in the absence of G418 to prevent possible toxic effects of G418 from complicating the analysis of cells in the –Doxy condition. Cells were assayed with the QuickCell Proliferation kit. For doxycycline-dependent clonal cell growth and reanimation, 500 MEF clone F cells were plated in a 6-well plate in the presence of 4 μg/mL doxycycline, 400 μg/mL G418, and 100 μg/mL hygromycin or hygromycin alone. All cells were grown with a 1:1000 dilution of Fungizone. At the end of the experiment, colonies were stained with crystal violet. To analyze the DNA sequence of the mutant hexanucleotide in stably transfected MEF cells (Figure 3), genomic DNA was isolated and the relevant sequence amplified by polymerase chain reaction and sequenced (Genewiz).

**Figure 4:**
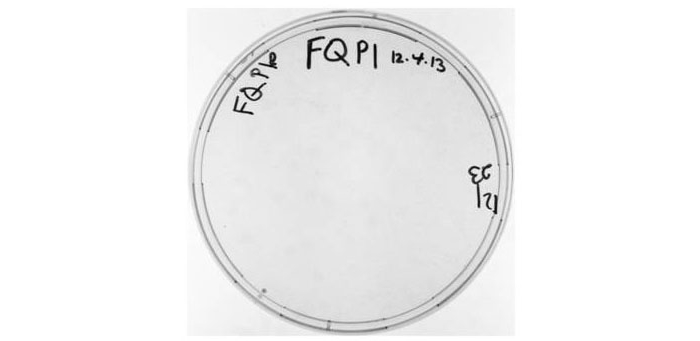
Adaptive mutation pilot experiment for cell growth treatment methodology. Lack of growth in small-scale pilot experiment demonstrates that system is working as expected, without significant nonspecific clonal growth.

To summarize, in this experimental design, MEF cells would be stably transfected with a plasmid (pRITAGKHexa) (Figure 1) expressing TAg from a doxycycline-inducible promoter, with a floxed wild-type polyA cassette and a downstream mutated polyA site (Figures 1-3). Cells would then be cotransfected with a plasmid expressing a floxed hygromycin-thymidine kinase (Hygro-TK) expression cassette; TK expression activates the prodrug ganciclovir, killing any cells retaining the Hygro-TK cassette. The purpose of ganciclovir selection in our system is to ensure that the only cells subjected to the experimental conditions are those that have successfully undergone CRE-Lox excision of the relevant floxed DNA sequences. Thus, the first steps of the main experimental protocol would be as follows. Cells would be infected with an adenovirus expressing CRE recombinase, which would remove both the wild-type polyA cassette from the integrated pRITAGKHexa plasmid and the Hygro-TK cassette from the integrated Hygro-TK plasmid. Cells would be plated out in replicates, with or without doxycycline, and with ganciclovir, which would kill any cells retaining the Hygro-TK cassette. Therefore, cells not infected with the adenovirus and that have not undergone CRE-Lox excision of floxed DNA sequences would be removed from the cell population. This is an important step because any cells not infected with CRE adenovirus would efficiently grow in the presence of doxycycline even without countermutation of the downstream polyA site. Thus, in noninfected cells, the continued presence of the floxed upstream wild-type polyA cassette would result in efficient 3′ end processing, high TAg expression, and clonal cell growth. Killing noninfected cells would eliminate the possibility of false positive results due to clonal growth of noninfected cells retaining the upstream polyA cassette. We would subsequently observe if infected cells treated with doxycycline form colonies suggesting the possibility of a reversal (countermutation) of the original polyA site mutation. Our preliminary data (Figure 4) demonstrate a lack of false positive results in 3 replicates of a pilot experiment infection.

The specific protocol utilized to generate these preliminary data (Figure 4) was as follows. A total of 150,000 MEF clone F cells were plated in a 6-well plate in the presence of 4 μg/mL doxycycline, 400 μg/mL G418, and 100 μg/mL hygromycin (F+). The next day, cells in media without the aforementioned added reagents were infected with Ad-CMV-CRE virus at a MOI of 1000 (assuming cell doubling). After 24-hr infection, cells were trypsinized and replated onto a 15-cm dish with 400 μg/mL G418 and 50 μM ganciclovir. After a further 24 hr, 4 μg/mL doxycycline was added to the media. Cells were incubated with doxycycline, G418, and ganciclovir (with regular changing of media) until the end of the experiment; at the end of the experiment (19 days postreplating), colonies were stained with crystal violet. All cells were grown with a 1:1000 dilution of Fungizone except during the 24-hr virus infection.

### Polyadenylation Site Mutations

A number of different mechanisms are postulated to lead to an adaptive response affecting gene expression: point mutations (eg, via base tautomerism), frameshift mutations, insertions and deletions, and epigenetic alterations. Although these possibilities can be potentially explained by classical mechanisms, we have a particular interest in evaluating the potential for a quantum biological explanation for adaptive mutation phenomena. The possibility of a quantum mechanism has informed our choice of experimental design, which is based on reversible mutation of polyadenylation (polyA) site signals.

In our experimental system, we would evaluate a polyA site mutation that can be reversed via base tautomerism to wild-type functional sequence. Thus, in the proper environment, a change in the cell state becomes irreversible due to clonal growth; this cell state is associated with a particular DNA sequence that allows for gene expression resulting in clonal growth. For example, consider cell state A_1_ characterized by a DNA sequence (ie, the original mutant polyA site) that does not allow for cell growth. In contrast, cell state A_2_ is characterized by the countermutation of the polyA site that restores TAg expression and allows for clonal cell growth. In the proper environment (presence of doxycycline), cells in state A_2_ undergo clonal growth, which is an irreversible change allowing cell state A_2_ to be observed. Because cell state A_2_ is associated with the DNA sequence allowing for cell growth, that sequence (of the polyA site) would also be observed when cellular DNA is assayed.

The specific mutations to be evaluated in this study are supported by Cooper’s [31-33] analysis of T4 bacteriophage mutation data. Cooper identified G and C bases as particularly sensitive to exist as a lowest-energy state consisting of a linear combination (ie, superposition) of G-C isomers [31-33]. Thus, the polyA site hexanucleotide sequences would incorporate 1, 2, or 3 mutant bases compared to the wild-type consensus sequence (AATAAA); these mutant sequences are AACAAA, AACAAG, or AACAGG. We chose to use base C in position 3 and base G in positions 5 and 6 because (1) mutations in these positions repress 3′ end processing [30] and (2) C to T and G to A conversions via base tautomerism [31-33] can restore the wild-type AATAAA hexanucleotide sequence. Thus, for a hexanucleotide sequence of AACAAA, the DNA template strand is TTGTTT; with RNA transcription, the mutant polyA site produced is AACAAA. However, if the G of the template strand is in the enol form (base tautomerism), it can base-pair with U resulting in wild-type AAUAAA sequence in the transcript. This would, in turn, restore wild-type 3′ end processing, TAg gene expression, and clonal cell growth. The mutation can also be reversed through DNA replication. Again considering the DNA sequence AACAAA, the complementary DNA strand is TTGTTT; the G in the enol form would base-pair with T restoring AATAAA. The same process can occur with G in positions 5 and 6 of the hexanucleotide sequence (AACAAG or AACAGG); thus, imino C in the complementary strand would base-pair with A, again restoring AATAAA.

Polymerase chain reaction (PCR) analysis, which has been used to probe DNA quantum superposition in vitro [34], also supports utilizing base tautomerism as the underlying rationale for the specific mutations to be used in our study. A study of the effects of primer-template mismatch on PCR efficiency determined that tautomeric G-T and C-A mismatches minimally affect PCR amplification; other types of base pair mismatch diminish amplification to a far greater extent [35]. Although that PCR study did not focus on molecular mechanisms, the findings are consistent with quantum-mediated base substitution being amplified by the PCR amplification process. Successful PCR amplification may be a “potential well” that “captures” base tautomerism (enol G and imino C) resulting from the linear combination of G-C isomers [31-33].

With respect to our pilot experiments, the triple mutation AACAGG exhibits the greatest inhibition of 3′ end processing and the greatest reduction in gene expression compared to wild-type AATAAA [30] (Figure 2). Therefore, we have generated MEF cell clones containing a version of pRITAGKHexa with a triple-mutant polyA site AACAGG (see preliminary data).

### Main Experimental Protocols: Fluctuation and Reconstruction Tests

The original Luria-Delbruck bacterial fluctuation test [36] was modified for use in mammalian cells [37-40] and can be used to distinguish between random and adaptive mutations. First, multiple samples from a single large culture (SLC) of the stably transfected MEF cells would be infected with Ad-CRE virus, plated out, and tested for colony growth as described previously. Then, separate multiple replicates (SMR) of the MEF cells would be produced; each replicate would be infected with Ad-CRE, plated out, and evaluated for colony formation. Cells would then be treated with doxycycline; as controls, we would culture 1 set of cells without doxycycline and this control should exhibit no clonal growth.

For both the SLC and SMR samples, numbers of resulting colonies from each set of replicates would be evaluated for the variance vs the mean. For the SLC samples, because the multiple samples come from the same single, original MEF cell culture, the variance would be similar to the mean with respect to number of colonies observed. However, for the SMR samples, each replicate is derived from an independent expansion of the MEF cell clone. Therefore, the relationship between the variance and the mean of colonies from these multiple independent replicates would depend on the type of mutation. For the SMR samples, a large variance vs mean would suggest random (and preexisting) mutation. The explanation for this finding would be that random mutations occur in the replicates at different times during cell culture expansion, so that each replicate would have a markedly different number of mutation-containing cells; hence, this would result in a large variance in colony formation. In contrast, a variance approximately equal/similar to the mean would suggest adaptive mutation. The explanation for this finding is that each replicate culture starts the experiment in the same condition of having approximately zero cells with mutation and the selective pressure induces the adaptive mutation at the same rate in each replicate culture. Thus, the number of colonies formed would be similar between cultures, resulting in a variance similar to the mean [36].

Reconstruction tests (reviewed in [17]) would be performed to determine whether late-forming cell colonies are (1) the result of (random or adaptive) mutations that occurred during the selection process or (2) are slow-growing cells with random mutations that occurred before Ad-CRE infection and before doxycycline exposure. Thus, cells from late-appearing colonies would be isolated and replated, and the clonal growth experiment would be repeated. As controls, cells from early-appearing colonies would be similarly replated. We would determine the time required for cells to form visible colonies of the same size as those from the original experiment. If cells from the early-appearing and late-appearing colonies grow at approximately the same rate in the reconstruction test, this means that late-appearing colonies were due to late-appearing (ie, during selection) mutations. However, if cells from later-appearing colonies exhibit slower growth kinetics than cells from early-appearing colonies, this would suggest that these are colonies derived from cells with mutations preexisting the selective pressure and that these cells have an innate slower growth rate.

In addition to clonal growth assays, cell proliferation can also be measured with the Quick Cell Proliferation assay kit, which we have already used to measure doxycycline-dependent growth of stably transfected MEF cells (Figure 2). It allows for high-throughput analysis of cell growth at defined time points in a 96-well format and the reagent used in this kit has limited toxicity and does not stain cells. Therefore, subsequent to measurements of cell growth, cells can be available for isolation of nucleic acids/proteins.

### Evaluation of Possible Mutator Phenotypes and DNA/RNA/Protein Analyses

If adaptive mutation is observed in the aforementioned experiments, mechanism of action would be analyzed by (1) for undirected adaptive mutation, we would evaluate whether changes in the expression of error-prone vs high-fidelity DNA polymerases, as well as MMR proteins, influences the general mutation rate in cells under selective pressure and (2) for directed adaptive mutation, base tautomerism resulting in base substitution mutation would be analyzed by DNA sequencing.

Evaluation of nonspecific adaptive mutations (eg, the “mutator phenotype”) would be as follows. First, reversible immortalization of MEF cells has not been shown to lead to random mutations allowing for growth because cells in the absence of TAg expression remain quiescent until TAg expression is restored [29]. Therefore, mutations that allow for cell growth in a manner independent of TAg expression have not been observed in previous studies of MEF cell reimmortalization. Second, we would determine polyA site sequence data for the MEF cell clones. If cell growth is observed even in the presence of the original inactivating mutation, this would suggest that an unknown, likely undirected, mutation activated clonal cell growth. However, if all MEF clones exhibit the predicted countermutation of the polyA site, this would support the directed mutation mechanism. Third, we would conduct hypoxanthine phosphoribosyltransferase (HPRT) assays, a commonly used method to determine increased mutation rates. Cells resistant to 6-thioguanine (6-TG) result from a random mutation in the *HPRT* gene, and any increase in 6-TG resistant cell growth is indicative of an increased undirected mutation rate. Cells should not exhibit enhanced resistance to 6-TG if directed mutation restores the wild-type polyA site sequence. However, if countermutation at the target polyA site is the result of an undirected increase in the mutation rate, then enhanced 6-TG resistance likely will be observed.

If undirected adaptive mutation is observed, we would evaluate whether the mechanism of mutation is through upregulation of error-prone DNA polymerases and downregulation of high-fidelity DNA polymerases and MMR proteins as has been reported in a prostate cancer cell line [16]. Protein lysates would be isolated from expanded cell colonies, and Western blot analysis performed to measure levels of (1) error-prone DNA polymerase (Pol ι), (2) high-fidelity DNA polymerases (Pol δ and Pol ε), and (3) MMR proteins (MSH6, PMS1, PMS2, and MLH1), as previously described [16].

If directed adaptive mutation is observed, nucleic acid sequence analyses would contribute to our understanding of the base tautomerism mechanism by which the mutation is generated. DNA and RNA would be isolated from cell colonies and/or from cells analyzed with the Quick Cell Proliferation assay kit. PCR (DNA) and reverse-transcription PCR (RNA) would be performed to amplify the target polyA region, followed by sequencing of the PCR products. Thus, we would determine whether cells exhibit reversal of the polyA site mutation. In addition, protein lysates would be isolated from expanded clonal growth and TAg protein expression would be measured by Western blot analysis. We would also perform PCR to confirm that the CRE-mediated excision took place and that the wild-type polyA cassette was removed from the TAg expression plasmid.

### Proposed Expected Results

Our primary expectation is that directed adaptive mutation would be observed and that the predicted base substitution mechanism would be confirmed by sequencing. We predict that after Ad-CRE infection and the addition of doxycycline, the cells will remain quiescent for a period of time and then commence clonal cell growth. It is possible that no adaptive mutation would occur because either (1) no clonal growth will occur or (2) growth will occur, but will be consistent with random mutation. Another possibility is that the undirected mutator phenotype form of adaptive mutation will be observed. If this occurs, we expect to find altered expression of error-prone vs high-fidelity DNA polymerases and MMR proteins [16]. It is possible that the triple mutation construct will present a mutation threshold that is too difficult to overcome via reverse mutation. In the event that no reversion of mutation occurs with the triple mutation, we would repeat the experimental design with the single and double mutation constructs (see discussion of polyA mutations).

### Alternative Approaches

One alternative approach to evaluate the possibility of directed adaptive mutation in mammalian cells is to use a frameshift mutation model in which a change in base sequence can confer resistance to a cytotoxic selective agent, thus allowing for cell growth/colony formation. We would use a plasmid that contains a thymidine kinase-neomycin resistance (TK-Neo) fusion gene and a sequence insert in the TK cassette that causes the subsequent Neo sequence to be out of frame for protein translation [41,42]. Frameshift mutation would alter the elongated sequence insert and restore Neo expression, resulting in resistance to the selection agent G418 [41,42]. The plasmid to be used for these experiments also has a constitutively expressed hygromycin selection cassette; thus, the plasmid can be stably transfected into mammalian cells. Cells would be exposed to a killing concentration of G418 and only cells with a frameshift mutation would express TK-Neo and exhibit G418-resistant clonal growth. The experimental design at that point would be similar to that described previously for the MEF cell studies. If the basis-dependent selection hypothesis is correct, then the frequency of mutation would be greater in the presence of the selective agent. Further, if we put another constraint on cell growth (eg, presence of serum in the tissue culture medium), then we would expect greater rates of specific mutation in the targeted TK-Neo sequence in the presence of the environmental condition that allows for cell growth (ie, presence of serum).

Another possible experimental design has been described elsewhere [27] and uses induction of bacteriophage lambda from the lysogenic to lytic form. This is a prokaryotic system and is, therefore, not an alternative approach for (eukaryotic) mammalian cells. Nevertheless, this prokaryotic approach may be useful in identifying directed adaptive mutation in a system well suited for rapid high-throughput analyses. Thus, for example, we can consider mutations that render the prophage unable to switch to the lytic pathway. The first mutation could be a temperature-sensitive mutation in a regulatory gene, such as cI repressor, whereas the second mutation would be in a gene required for the next step in induction (eg, antiterminator N). The first mutation would be controlled as part of the experiment (eg, changing the temperature to influence cI expression), whereas alteration in the second mutation (ie, countermutation restoring wild-type sequence and gene expression) would occur in a manner not directly influenced by the experimenter. According to the hypothesis of basis-dependent selection, reversion of the second mutation to active wild type would occur more frequently when the cI repressor is inactivated by the temperature-sensitive mutation. In other words, a mutation that allows for lytic induction would occur more frequently in circumstances in which lytic growth is permissible (no cI repressor activity).

### Purpose of Paper

Directed adaptive mutations arise when selective pressure induces targeted mutations that specifically influence the adaptive response; identification of directed adaptive mutation would have profound practical significance for a wide variety of biomedical problems, including disease development and resistance to treatment. The possibility of directed adaptive mutation has not been experimentally assessed due to the lack of an appropriate experimental system. We believe that this fundamental methodological deficiency is addressed by our proposed system; thus, this manuscript describes a possible experimental approach to determine whether directed adaptive mutation occurs in mammalian cells. This approach is based on our model of basis-dependent selection describing a quantum biological mechanism of adaptive mutation.

This project is currently inactive due to lack of funding; the data we have are preliminary, derived from a small number of pilot experiments. However, a major objective of early reports is to discuss projects that have, for whatever reason, not produced publishable data, but are worthy of discussion and analysis because of novel experimental designs, innovative protocols, and/or previously unexplored hypotheses. Thus, we outline the project’s rationale and experimental design to stimulate discussion and analysis, and lay the foundation for future studies in this field. Readers can identify strengths and weaknesses of our hypotheses, overall approach, experimental design, and specific research protocols. Thus, critical analysis from the scientific community may lead to improved methodologies for identifying adaptive mutation and for evaluating the validity of basis-dependent selection.

## Methods

### Plasmids

The pRITAGKHexa plasmids were constructed as follows. The plasmid pGKNeotpAlox2 (Addgene) was digested with *Eco*R1 and *Hin*dIII, Klenow treated, and religated to yield pGKDelta, lacking the neomycin gene. pRITAGK was constructed by ligating the *Nhe*I (Klenow-treated)-*Pac*one large fragment of pRITA with the *Nhe*I-*Pac*I small fragment of pRITA and the *Sac*I (Klenow-treated)-*Nhe*I fragment of pGK delta containing the floxed SV40 late polyA sites. pRITAHexa plasmids were constructed by ligating the *Bgl*II-*Pac*I large fragment of pRITAGK the *Nhe*1-*Bgl*II fragment of pRITAGK containing the floxed SV40 late polyA sites and the *Nhe*I-*Pac*I small fragments from constructs obtained from IDT Technologies, containing a cassette of wild-type (AATAAA) SV40 late polyA hexanucleotide sequences or one of the following mutations: AACAAA, AACAAG, or AACAGG. This formed plasmids pRITAGKHexa-WT, pRITAGKHexa-C, pRITAGKHexa-CG, and pRITAGKHexa-CGG, respectively. This cassette contains *Xho*I sites for screening and SP6 and T3 sites for sequencing analysis of the polyA cassette, distinguishing this polyA site from those in the floxed pGK sequences. To construct p3.1hTKΔNeo, the Hygro-TK plasmid from Alex Bortvin was digested with *Not*I to remove the hygromycin-thymidine kinase cassette. This was inserted into pcDNA3.1-, cut with *Not*I, and treated with calf intestinal alkaline phosphatase. To remove neomycin-resistance function, the bulk of the neomycin gene was removed from the resulting construct by digestion with *Bst*BI and *Nar*I, Klenow treatment, and religation.

### Stable Transfection: CRE-Lox Excision of Mixed Cell Population

The plasmids pRITAGKHexa-WT, pRITAGKHexa-C, pRITAGKHexa-CG, and pRITAGKHexa-CGG were linearized with *Pac*I and p3.1hTKΔNeo was linearized with *Mfe*I. Each of the pRITA-based plasmids was stably cotransfected by nucleofection into MEF cells along with p3.1hTKΔNeo and selection was performed with 400 μg/mL G418 and 150 μg/mL hygromycin.

MEF cells stably transfected with the triple-mutant (CGG) pRITAGKHexa plasmid and Hygro-TK plasmids were infected or mock infected with Ad-CRE adenovirus at a MOI of 1000. Cells exposed to Ad-CRE were treated with 50 μM ganciclovir to eliminate cells that were not infected and did not undergo CRE-Lox excision. Three days later, protein was isolated and Western blot analysis performed with anti-SV40 TAg and antiactin antibodies.

These cells were then subjected to clonal selection and individual clones were grown and tested as described subsequently for growth characteristics.

### Doxycycline-Dependent Cell Proliferation

MEF cells were plated out at 4000 cells/well in a 96-well plate with 4 μg/mL doxycycline, 400 μg/mL G418, and 100 μg/mL hygromycin or hygromycin alone. Cells were assayed with the QuickCell Proliferation kit (Biovision). For doxycycline-dependent clonal cell growth and reanimation, 500 MEF clone F cells were plated in a 6-well plate in the presence of 4 μg/mL doxycycline, 400 μg/mL G418, and 100 μg/mL hygromycin or hygromycin alone. All cells were grown with a 1:1000 dilution of Fungizone. At the end of the experiment, colonies were stained with crystal violet.

### Polymerase Chain Reaction and DNA Sequencing

PCR was performed with a Promega PCR core kit, with a hot start with the following parameters: 1 cycle of 95 °C for 6 minutes; 50 cycles of 95, 56, and 72 °C for 30 seconds each; and 1 cycle of 72 °C for 10 minutes. The following primers were used: X6 (CTCGAGAGGATTTAGGTGACACT) and RGKup (CAATACGCAAACCGCCTCTC). Sequencing of PCR products was performed by Genewiz with the P3 alternative sequencing protocol (custom unprocessed PCR sequencing).

### Adaptive Mutation Pilot Experiment

A total of 150,000 MEF clone F cells were plated in a 6-well plate in the presence of 4 mg/mL doxycycline, 400 μg/mL G418, and 100 μg/mL hygromycin. The next day, cells in media without the aforementioned added reagents were infected with Ad-CMV-CRE virus at a MOI of 1000. After 24-hr infection, cells were trypsinized and replated onto a 15-cm dish with 400 μg/mL G418 and 50 μM ganciclovir. After a further 24 hr, 4 μg/mL doxycycline was added to the media. Cells were incubated with doxycycline, G418, and ganciclovir (with regular changing of media) until the end of the experiment; at the end of the experiment (19 days postreplating), colonies were stained with crystal violet. All cells were grown with a 1:1000 dilution of Fungizone except during the 24-hr virus infection.

## Results

### Preliminary Data Characterizing the Experimental Design

#### Plasmids, Stable Transfection, and CRE-Lox Excision of Mixed Cell Population

MEF cells were stably transfected with the pRITAGKHexa plasmid (Figure 1) containing the downstream polyA site with the triple-mutant AACAGG hexanucleotide sequence, and cotransfected with the floxed Hygro-TK plasmid. After G418 and hygromycin selection, we performed a pilot experiment in which CRE was delivered by adenovirus (Ad-CRE), resulting in excision of the floxed sequences in the stably transfected plasmids. Cells were then treated with 50 μM ganciclovir, which killed those cells that did not undergo CRE-mediated excision of the floxed genes. The remaining living cells were those that were successfully infected with the Ad-CRE virus. Western blot analysis confirmed downregulated TAg expression because of inefficient 3′ end processing of the remaining mutant polyA site (Figure 2).

#### Doxycycline-Dependent Cell Proliferation

We then performed clonal selection to identify a stably transfected MEF cell clone that exhibits doxycycline-inducible expression with rapid cell growth in the presence of doxycycline and minimal growth in the absence of doxycycline. Clone F exhibited proliferation in the presence of doxycycline (Figure 2, gray squares) and exhibited minimal nonspecific proliferation in the absence of doxycycline (Figure 2, black diamonds).

Although proliferation assays are a reasonable screening approach to find an appropriate clone, the main metric for this study is clonal growth. Therefore, we performed clonal growth assays on clone F (Figure 2). In the absence of doxycycline (F–), we observed scattered individual cells or larger clusters of cells mostly exhibiting varied forms of senescent phenotypes, including very large flattened cells or elongated cells. These phenotypes are characteristic of senescent fibroblast cells. In the presence of doxycycline (F+), we observed fast-growing large and dense colonies of phenotypically normal fibroblasts; these types of colonies are the endpoint for a positive signal of clonal cell outgrowth. After 3 days cultured without doxycycline (F3), clone F cells could be reanimated to form colonies of phenotypically normal cells, similar to what is observed with F+. After 6 days cultured in the absence of doxycycline (F6), cells began to lose their ability to be efficiently reanimated; however, after exposure to doxycycline, a few colonies of dividing normal cells were observed. F3 and F6 samples exhibited a greater proportion of senescent cells and fewer dividing colonies compared to F+ as a consequence of the periods cultured in the absence of doxycycline (data not shown). Clone F cells are, as expected, sensitive to ganciclovir; thus, when cultured in the presence of both doxycycline and ganciclovir (FG), no colonies were observed (Figure 2).

#### Polymerase Chain Reaction and DNA Sequencing

Clone F was further characterized by sequencing the target polyA sequence from PCR-amplified genomic DNA, similar to what we planned for our experimental protocol. The expected mutant hexanucleotide sequence was observed (Figure 3).

### Adaptive Mutation Pilot Experiment

We subsequently conducted a pilot experiment of the basic adaptive mutation experimental protocol outlined in Figure 1. Given the number of cells used in this experiment (150,000 cells originally plated and allowed to grow overnight), no colonies were observed after approximately 3 weeks (Figure 4). This experiment was repeated twice with similar results (data not shown). These findings support the stringency of our experimental system. Thus, if the system were “leaky” (with cells that escaped both infection/CRE recombination and ganciclovir treatment), then cell colonies would likely have been observed given the number of cells plated. Further, these findings are also consistent with a very low frequency of putative adaptive mutation events because no clonal outgrowth was observed with 3 replicates of this pilot experiment. Therefore, to assay for adaptive mutation, a greater number of cells (eg, millions) will be required for assay in each replicate of the experiment.

### Summary

In general, clone F exhibits a number of properties suitable for our experimental design; however, the characterization of even more optimal clones likely would be required. For example, clone F exhibits some degree of doxycycline-independent growth after plating (Figure 2), similar to several other clones tested (data not shown); in contrast, a fully optimized MEF cell clone would exhibit absolutely no cell growth in the absence of doxycycline.

## Discussion

### Summary

This paper is an early report of a proposed project aimed at evaluating the hypothesis of basis-dependent selection as a mechanism generating directed adaptive mutation in a mammalian cell model system. We have outlined the experimental design, expected results, and have presented preliminary data from pilot experiments characterizing the fundamentals of the proposed system. Thus, readers can identify strengths and weaknesses of our experimental design, leading to improved methodologies for identifying adaptive mutation and for evaluating the validity of basis-dependent selection.

### Relevance of Model Systems

One potential criticism of our experimental approach is that model systems are artificial and do not reflect the full complexity of endogenous genes and endogenous microenvironments. However, we believe that a model system approach is the most efficient initial screening method for identifying a heretofore-unknown biological mechanism. The complexity of endogenous systems introduces many variables that would complicate the evaluation and optimization of our methodology, and would also complicate the definitive identification of directed adaptive mutation. By reducing variables, model systems more effectively allow for the identification of novel biological processes, particularly those suspected to occur at a low frequency. In vitro models are often used to characterize complex biological systems and generally have significant predictive value when findings are subsequently applied in vivo [43]. Thus, the use of model systems for the initial characterization of directed adaptive mutation is warranted.

### Further Possible Future Studies

If our proposed project produced findings consistent with undirected adaptive mutation, we would subsequently evaluate therapeutic approaches to reduce the increased mutation rate. For example, siRNA-mediated knockdown of error-prone DNA polymerases, and/or exogenous overexpression of high-fidelity DNA polymerases and MMR proteins could restore a wild-type cell phenotype. If the findings were consistent instead with directed adaptive mutation, we would then determine the general applicability of the phenomenon by evaluating other experimental systems. For example, we would investigate whether directed adaptive mutation contributes to cancer initiation/progression or whether cancer cells use directed adaptive mutation to evade anticancer therapy. Evaluating adaptive mutation during development of a multicellular organism (eg, earliest stages of Drosophila development) is another potential future approach. We would also assess whether targeted alteration of the cellular microenvironment can induce specific, therapeutically favorable mutations (eg, those that increase sensitivity to treatment or which enhance terminal differentiation of neoplastic cells).

### Significance

Adaptive mutation has significance for a wide variety of biomedical problems, including carcinogenesis, development of resistance to treatment for cancer and other diseases, and modulation of cell/tissue plasticity in experimental and/or clinical approaches for gene therapy, stem cell therapies, or tissue regeneration. Directed adaptive mutation could force a reevaluation of certain drug-based therapeutic strategies. For example, our model of adaptive mutation [27] requires that cells have sufficient time to “explore” the space of possibilities. Thus, to prevent directed adaptive mutation, it would be important to use cytotoxic methods to kill cells quickly [27]. Conversely, longer-duration treatments that allow cells to explore the adaptive possibilities could be used to promote clinically favorable mutations (eg, those causing differentiation and/or apoptosis). There is also practical importance in distinguishing directed vs undirected adaptive mutation [27]. Understanding directed adaptive mutation may lead to approaches that use targeted manipulation of the microenvironment to prevent or induce specific mutations and consequent changes in gene expression. In contrast, therapeutic approaches for undirected adaptive mutation would be limited to suppressing mutation rates.

**Figure 5:**
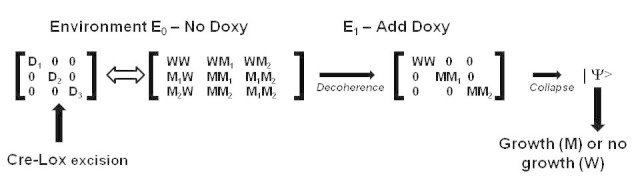
Quantum representation of the experimental design resulting in adaptive mutation. The “D” states represent preferred cell states in an environment lacking doxycycline and after CRE-Lox excision. The “W” states represent the cell state that is wild type from the perspective of the original inactive polyA site, whereas the “M” states represent cell states in which mutation (eg, the polyA site) allows for cell growth in the presence of doxycycline.

Findings from our proposed experimental system may also have implications for the theory of basis-dependent selection previously described [27]. Thus, for the sake of theoretical evaluation, a quantum mechanical density matrix formalism describing the experimental design and potential results is shown in Figure 5.

## Multimedia Appendix 1

Powerpoint presentation of key concepts of basis-dependent selection.

## Abbreviations

6-TG: 6-thioguanine
GFP: green fluorescent protein
HPRT: hypoxanthine phosphoribosyltransferase
MEF: mouse embryo fibroblast
MOI: multiplicity of infection
PCR: polymerase chain reaction
SLC: single large culture
SMR: separate multiple replicates
SV40: simian virus 40
TAg: large T antigen
