# Supplementary material for: Experimental Design to Evaluate Directed Adaptive Mutation in Mammalian Cells
Source: JMIR Res Protoc. 2014 Dec 9;3(4):e74. doi: 10.2196/resprot.3860 (PMC4275479; doi:10.2196/resprot.3860)
Supplement: Supplementary file 1 [file resprot_v3i4e74_app1.ppt]

## Slide 1
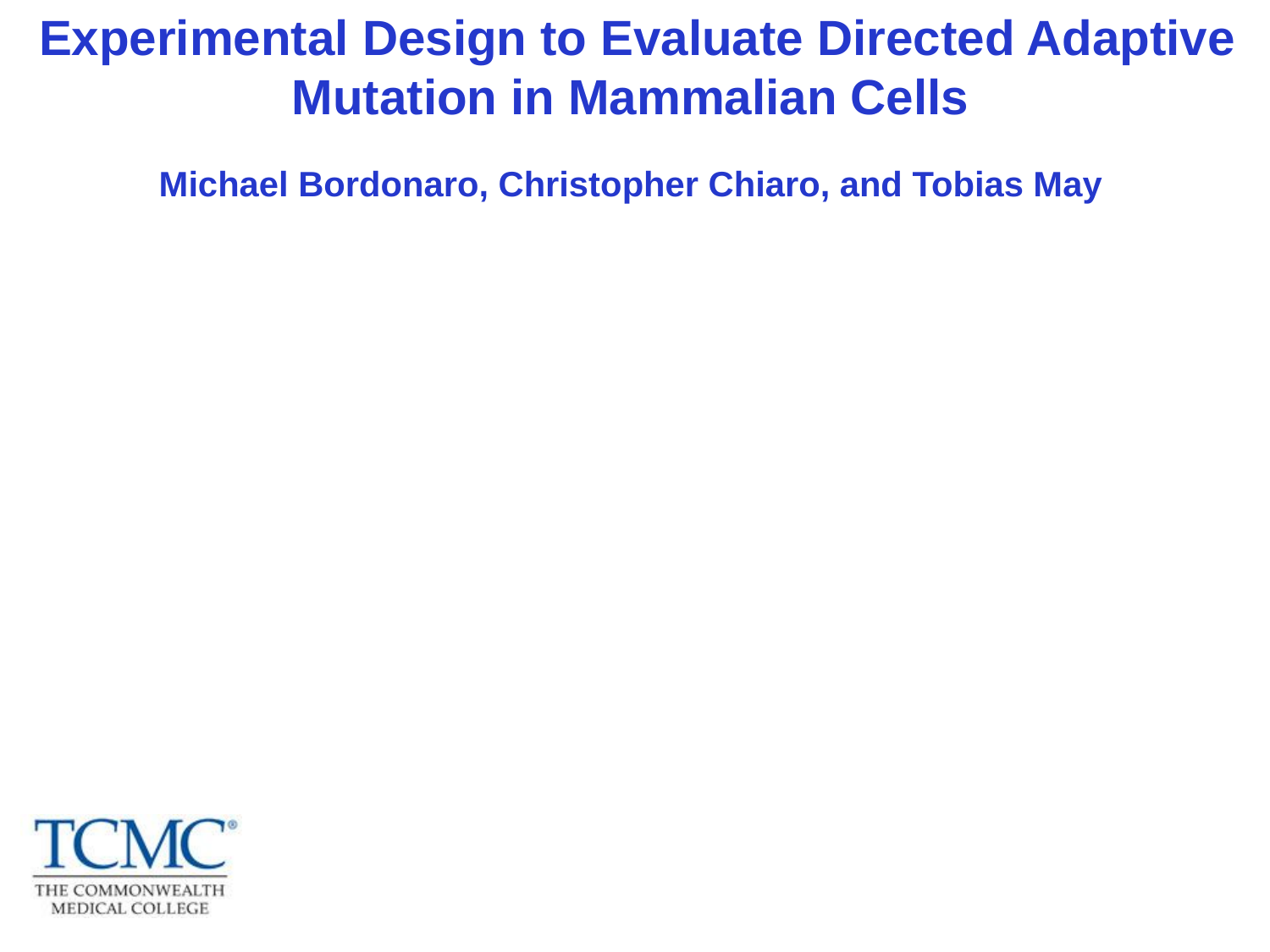

Experimental Design to Evaluate Directed Adaptive Mutation in Mammalian Cells
Michael Bordonaro, Christopher Chiaro, and Tobias May

## Slide 2
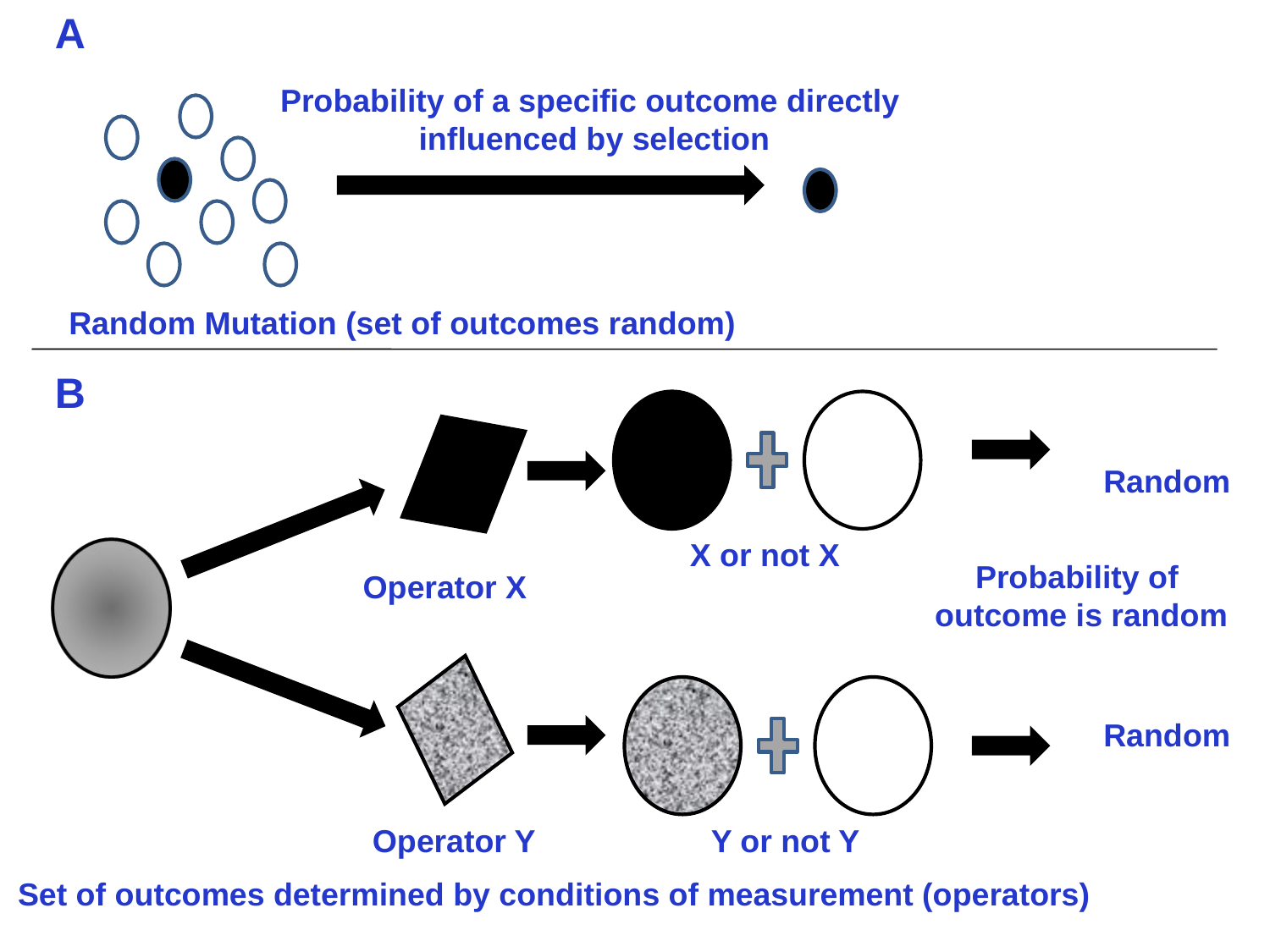

A
Probability of a specific outcome directly
influenced by selection
Random Mutation (set of outcomes random)
B
Random
X or not X
Probability of
outcome is random
Operator X
Random
Operator Y
Y or not Y
Set of outcomes determined by conditions of measurement (operators)

## Slide 3
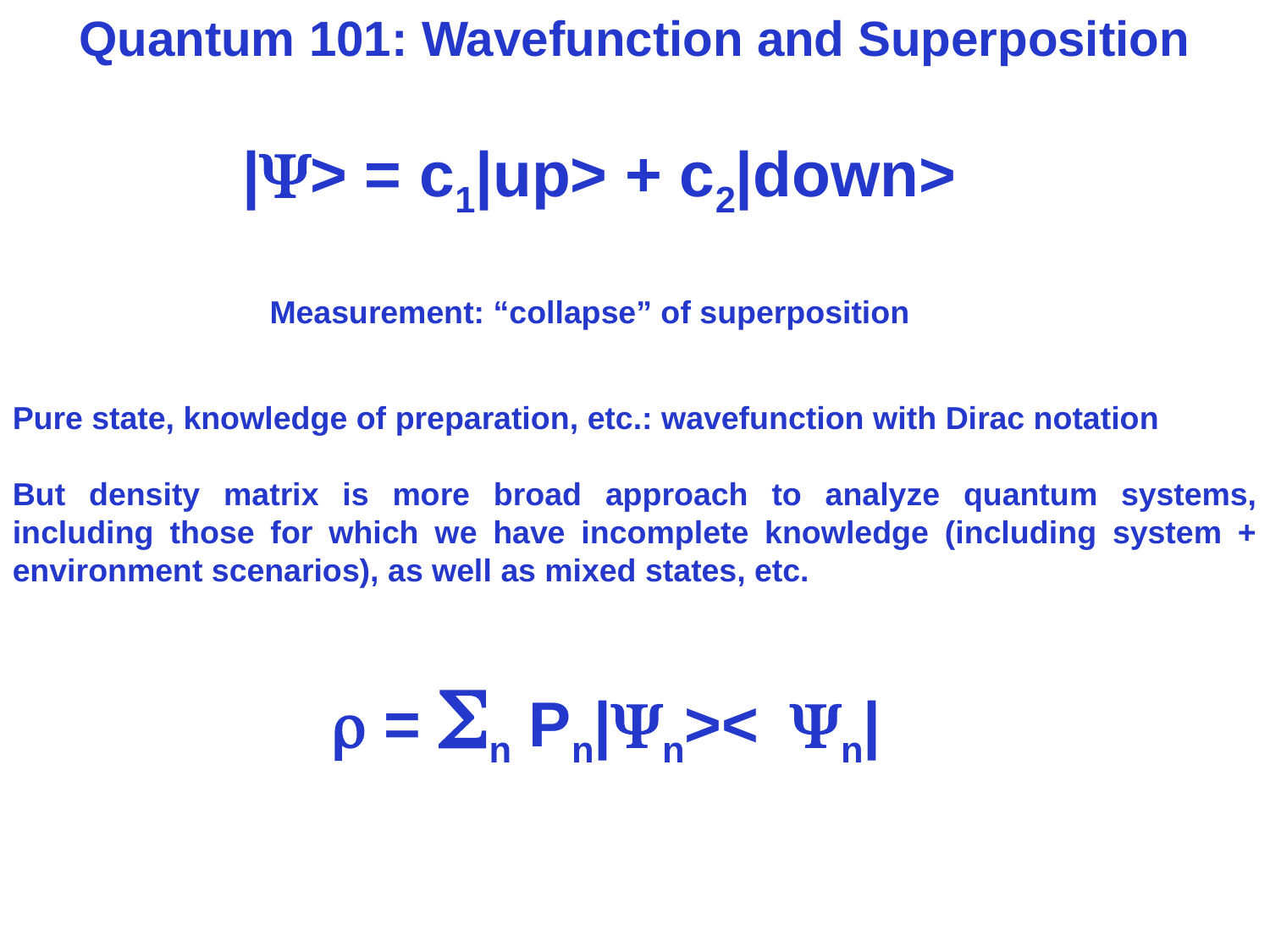

Quantum 101: Wavefunction and Superposition
|> = c1|up> + c2|down>
Measurement: “collapse” of superposition
Pure state, knowledge of preparation, etc.: wavefunction with Dirac notation
But density matrix is more broad approach to analyze quantum systems, including those for which we have incomplete knowledge (including system + environment scenarios), as well as mixed states, etc.
 = n Pn|n>< n|

## Slide 4
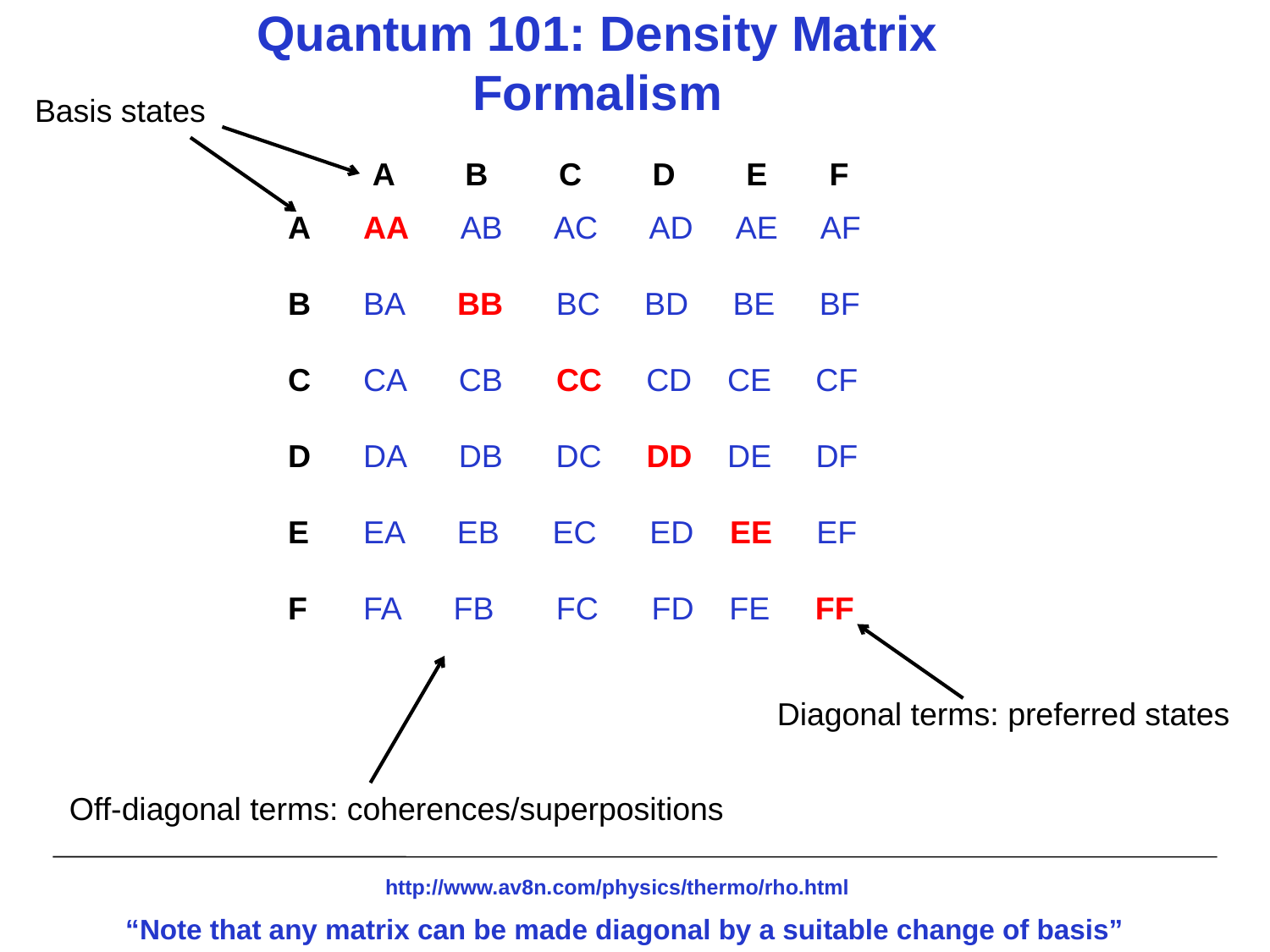

Quantum 101: Density Matrix Formalism
Basis states
A B C D E F
A
B
C
D
E
F
AA AB AC AD AE AF
BA BB BC BD BE BF
CA CB CC CD CE CF
DA DB DC DD DE DF
EA EB EC ED EE EF
FA FB FC FD FE FF
Diagonal terms: preferred states
Off-diagonal terms: coherences/superpositions
http://www.av8n.com/physics/thermo/rho.html
“Note that any matrix can be made diagonal by a suitable change of basis”

## Slide 5
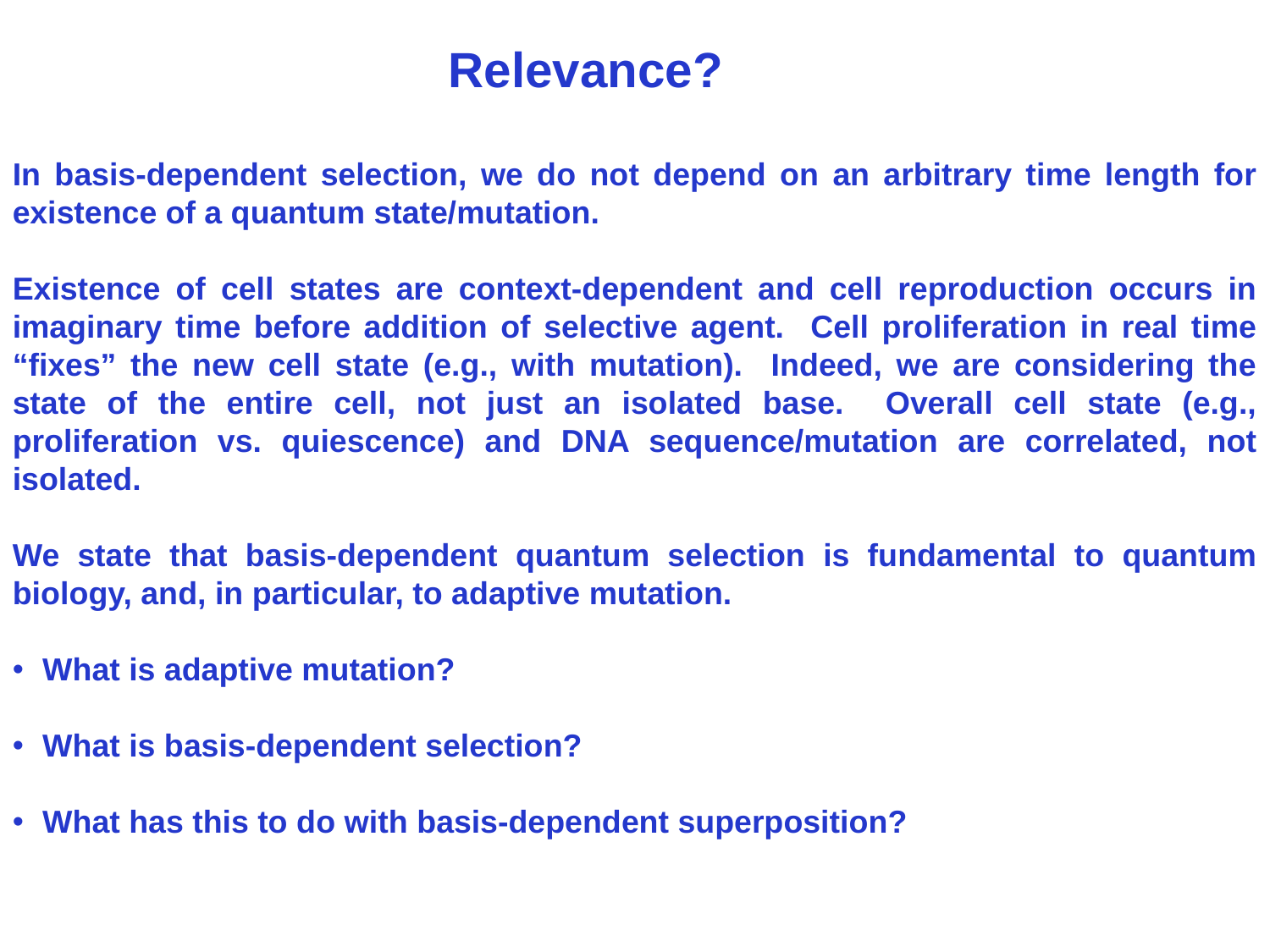

Relevance?
In basis-dependent selection, we do not depend on an arbitrary time length for existence of a quantum state/mutation.
Existence of cell states are context-dependent and cell reproduction occurs in imaginary time before addition of selective agent. Cell proliferation in real time “fixes” the new cell state (e.g., with mutation). Indeed, we are considering the state of the entire cell, not just an isolated base. Overall cell state (e.g., proliferation vs. quiescence) and DNA sequence/mutation are correlated, not isolated.
We state that basis-dependent quantum selection is fundamental to quantum biology, and, in particular, to adaptive mutation.
What is adaptive mutation?
What is basis-dependent selection?
What has this to do with basis-dependent superposition?

## Slide 6
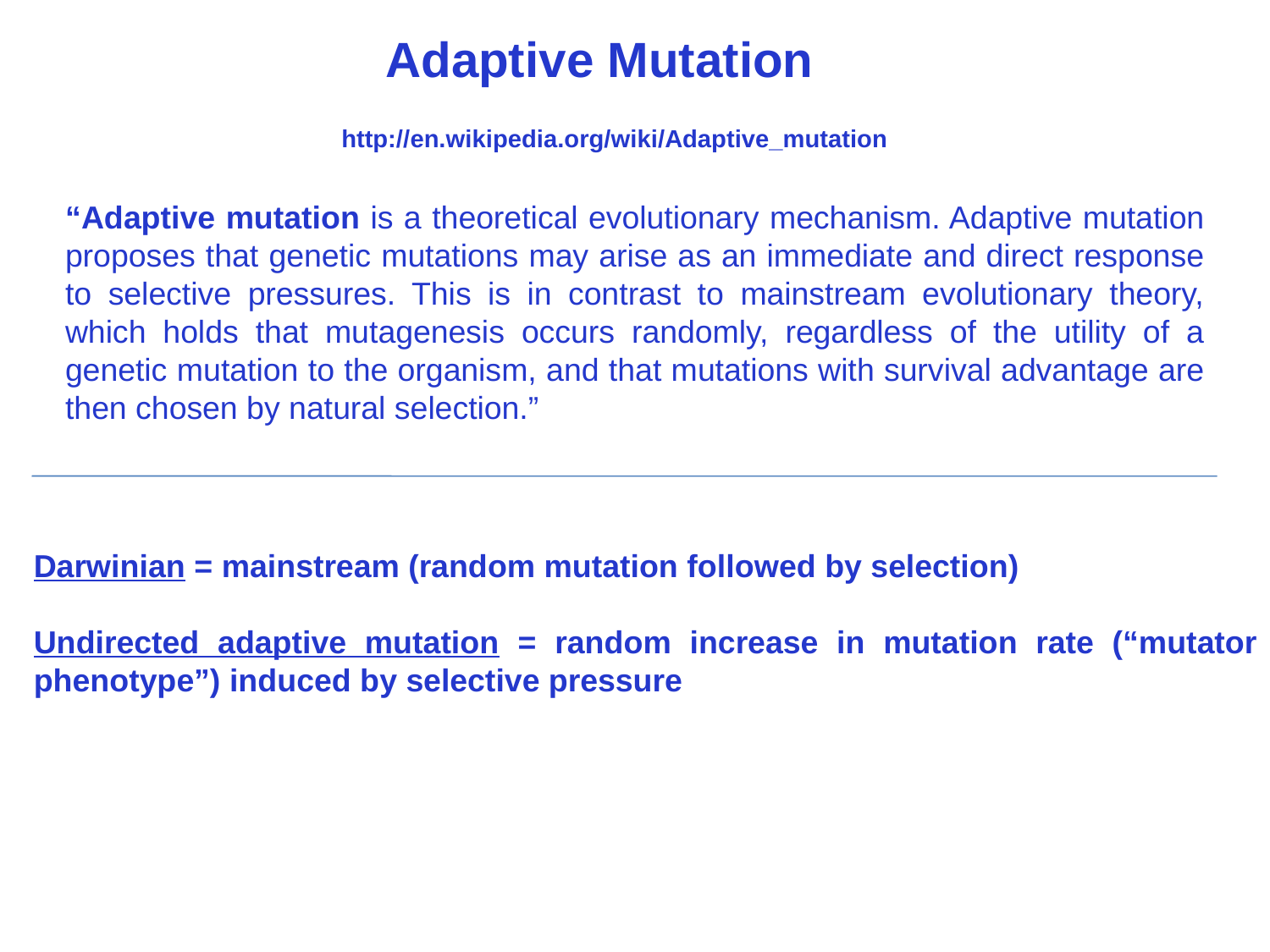

Adaptive Mutation
http://en.wikipedia.org/wiki/Adaptive_mutation
“Adaptive mutation is a theoretical evolutionary mechanism. Adaptive mutation proposes that genetic mutations may arise as an immediate and direct response to selective pressures. This is in contrast to mainstream evolutionary theory, which holds that mutagenesis occurs randomly, regardless of the utility of a genetic mutation to the organism, and that mutations with survival advantage are then chosen by natural selection.”
Darwinian = mainstream (random mutation followed by selection)
Undirected adaptive mutation = random increase in mutation rate (“mutator phenotype”) induced by selective pressure

## Slide 7
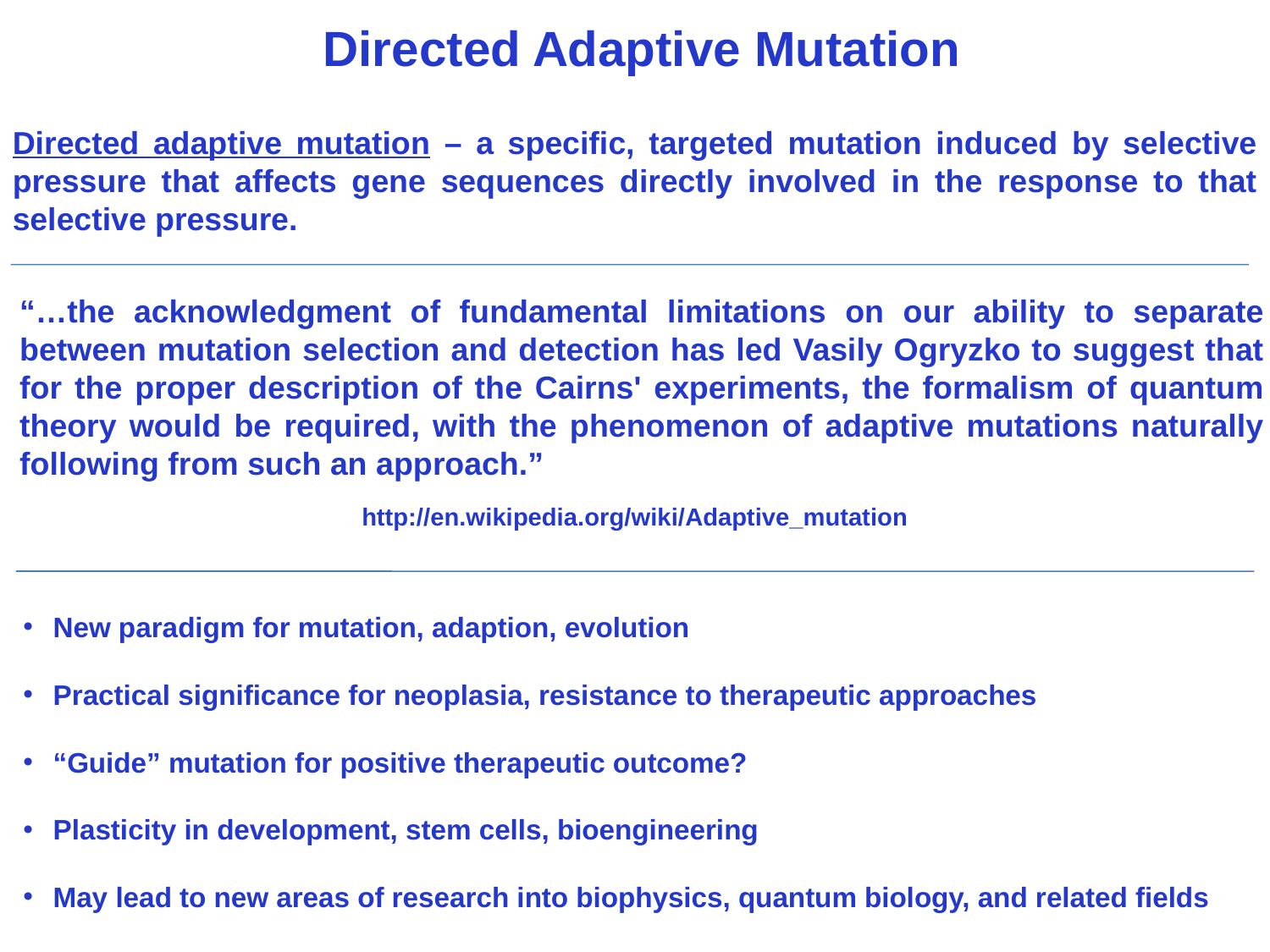

Directed Adaptive Mutation
Directed adaptive mutation – a specific, targeted mutation induced by selective pressure that affects gene sequences directly involved in the response to that selective pressure.
“…the acknowledgment of fundamental limitations on our ability to separate between mutation selection and detection has led Vasily Ogryzko to suggest that for the proper description of the Cairns' experiments, the formalism of quantum theory would be required, with the phenomenon of adaptive mutations naturally following from such an approach.”
http://en.wikipedia.org/wiki/Adaptive_mutation
New paradigm for mutation, adaption, evolution
Practical significance for neoplasia, resistance to therapeutic approaches
“Guide” mutation for positive therapeutic outcome?
Plasticity in development, stem cells, bioengineering
May lead to new areas of research into biophysics, quantum biology, and related fields

## Slide 8
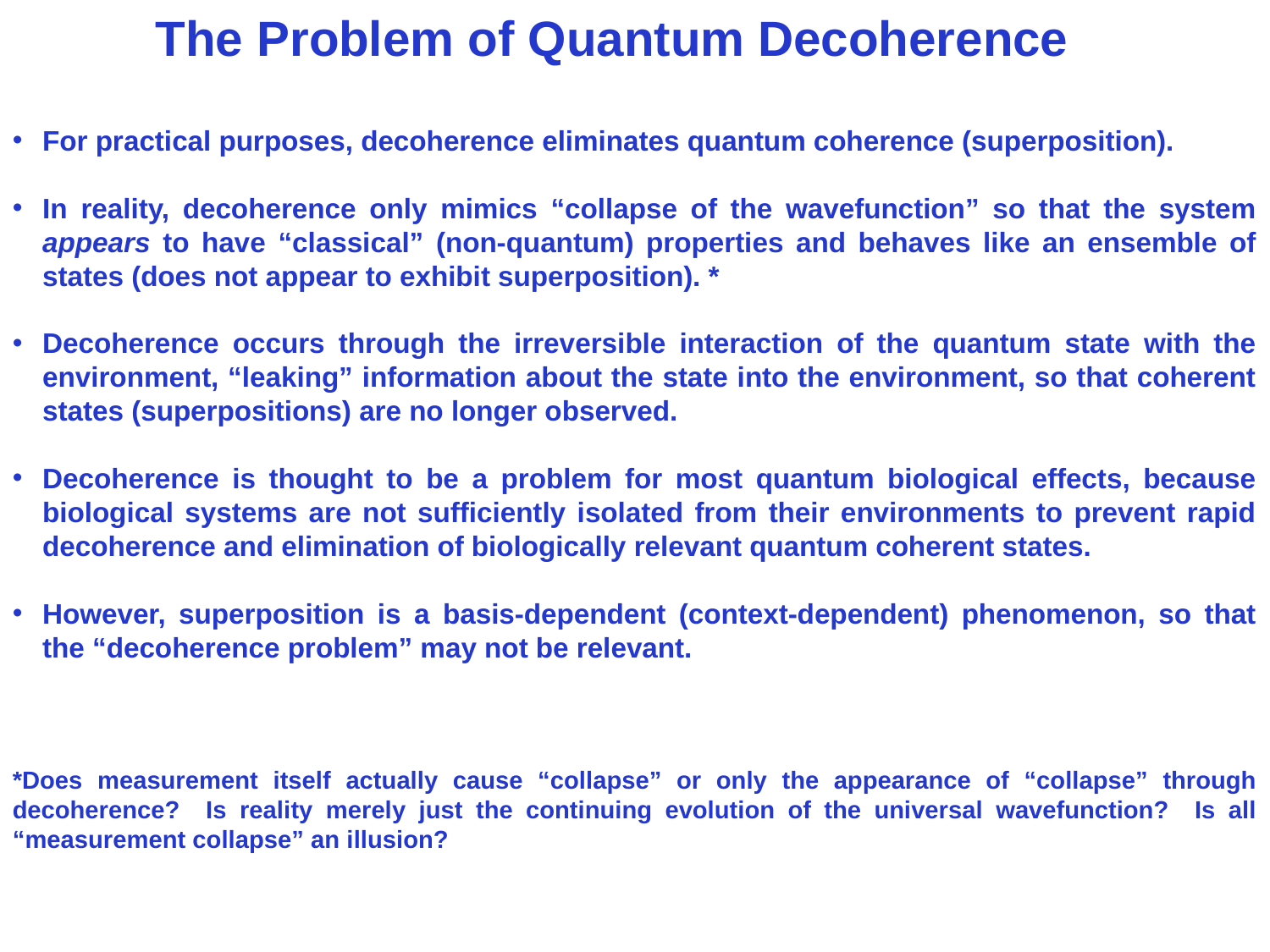

The Problem of Quantum Decoherence
For practical purposes, decoherence eliminates quantum coherence (superposition).
In reality, decoherence only mimics “collapse of the wavefunction” so that the system appears to have “classical” (non-quantum) properties and behaves like an ensemble of states (does not appear to exhibit superposition). *
Decoherence occurs through the irreversible interaction of the quantum state with the environment, “leaking” information about the state into the environment, so that coherent states (superpositions) are no longer observed.
Decoherence is thought to be a problem for most quantum biological effects, because biological systems are not sufficiently isolated from their environments to prevent rapid decoherence and elimination of biologically relevant quantum coherent states.
However, superposition is a basis-dependent (context-dependent) phenomenon, so that the “decoherence problem” may not be relevant.
*Does measurement itself actually cause “collapse” or only the appearance of “collapse” through decoherence? Is reality merely just the continuing evolution of the universal wavefunction? Is all “measurement collapse” an illusion?

## Slide 9
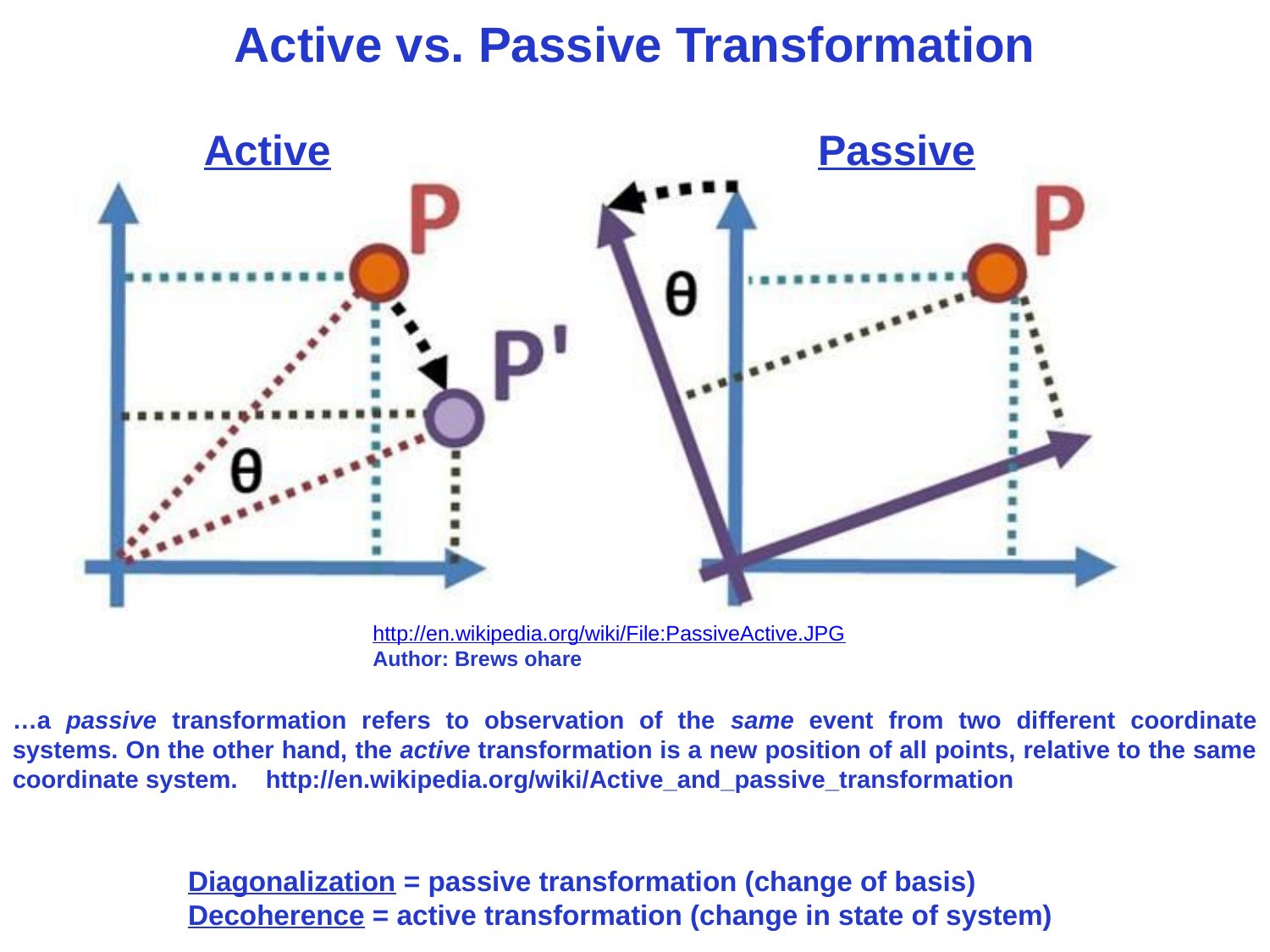

# Active vs. Passive Transformation
Active
Passive
http://en.wikipedia.org/wiki/File:PassiveActive.JPG
Author: Brews ohare
…a passive transformation refers to observation of the same event from two different coordinate systems. On the other hand, the active transformation is a new position of all points, relative to the same coordinate system. http://en.wikipedia.org/wiki/Active_and_passive_transformation
Diagonalization = passive transformation (change of basis)
Decoherence = active transformation (change in state of system)

## Slide 10
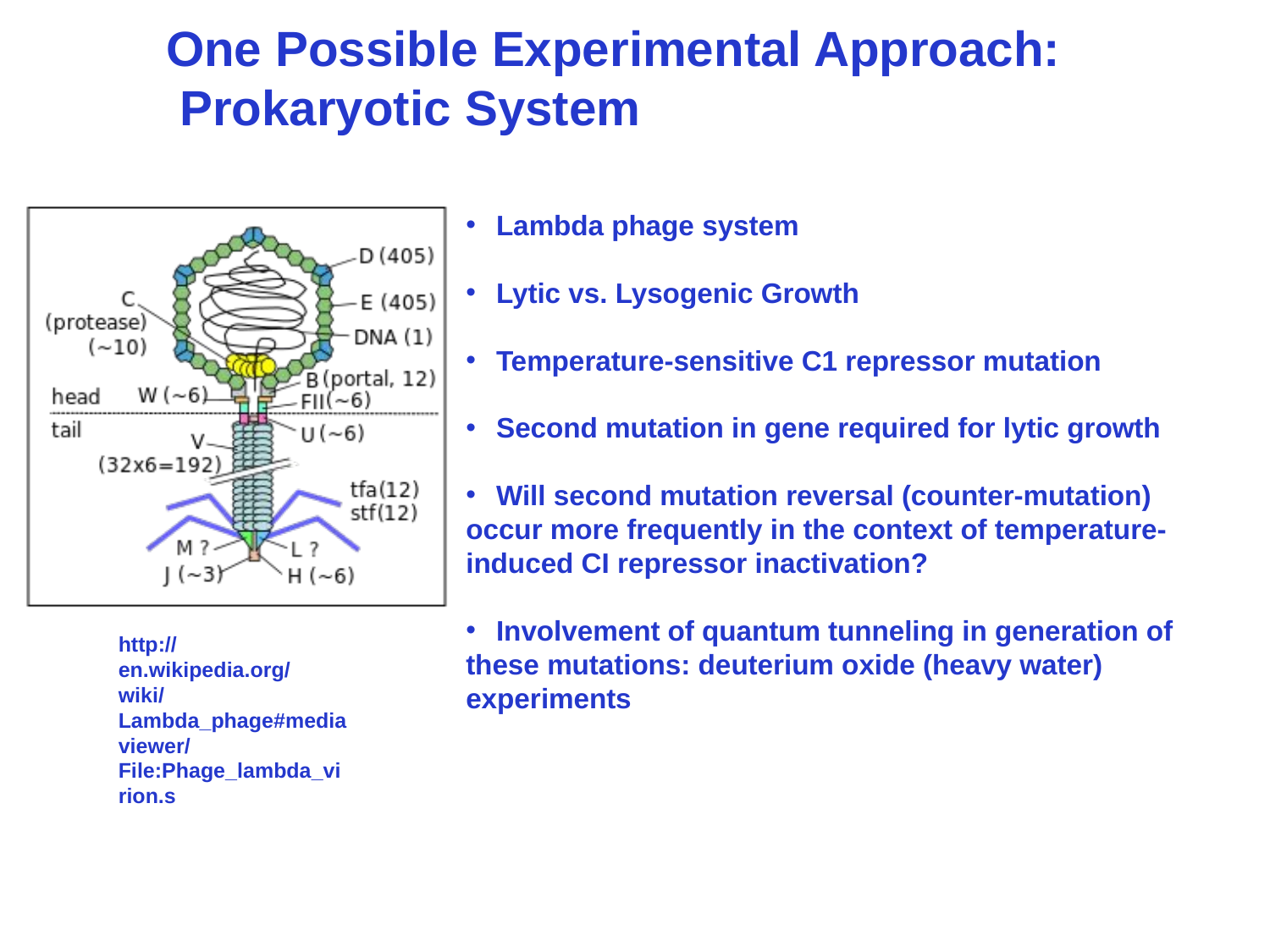

One Possible Experimental Approach:
 Prokaryotic System
Lambda phage system
Lytic vs. Lysogenic Growth
Temperature-sensitive C1 repressor mutation
Second mutation in gene required for lytic growth
Will second mutation reversal (counter-mutation)
occur more frequently in the context of temperature-
induced CI repressor inactivation?
Involvement of quantum tunneling in generation of
these mutations: deuterium oxide (heavy water)
experiments
http://en.wikipedia.org/wiki/Lambda_phage#mediaviewer/File:Phage_lambda_virion.s

## Slide 11
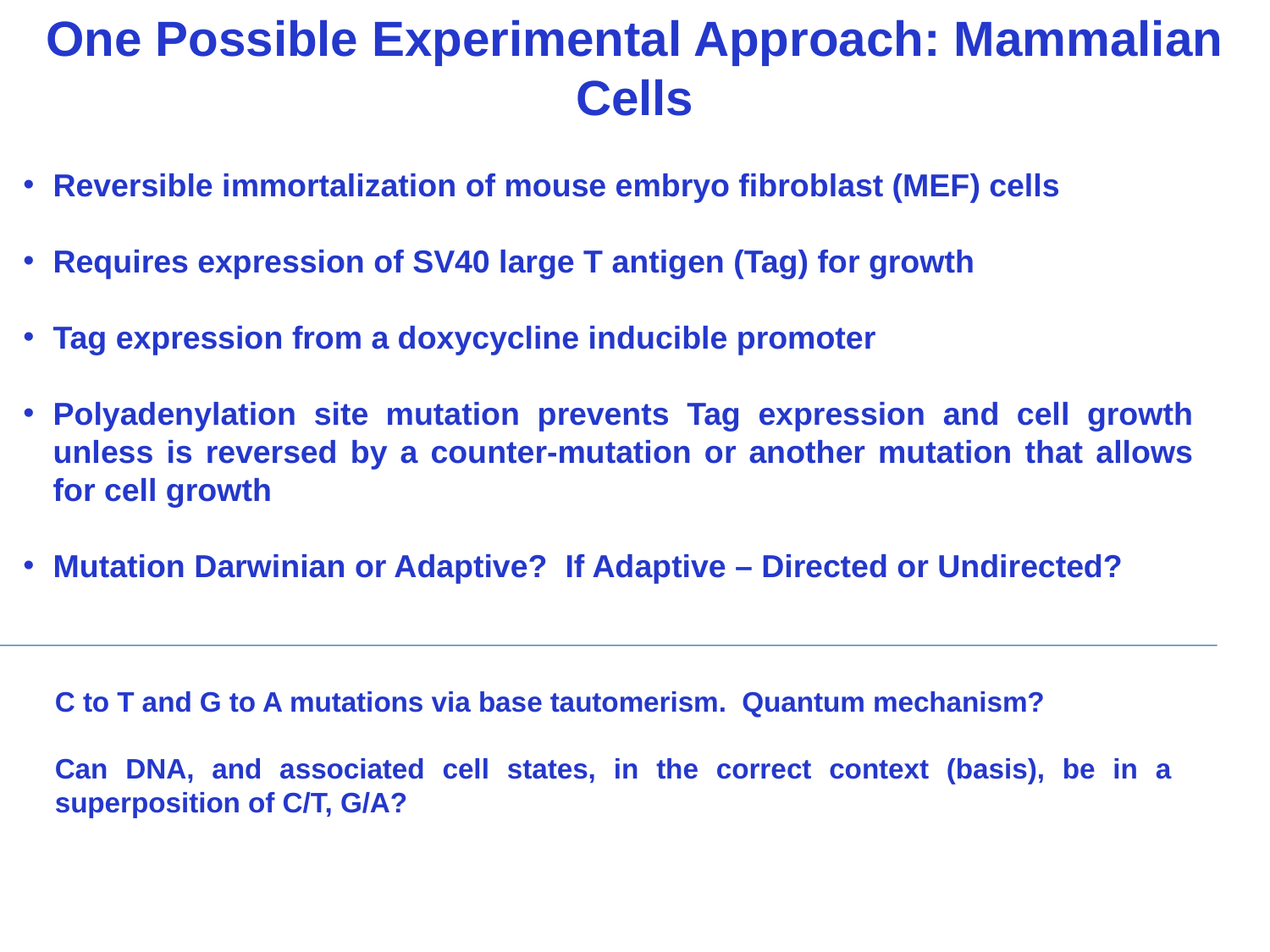

One Possible Experimental Approach: Mammalian Cells
Reversible immortalization of mouse embryo fibroblast (MEF) cells
Requires expression of SV40 large T antigen (Tag) for growth
Tag expression from a doxycycline inducible promoter
Polyadenylation site mutation prevents Tag expression and cell growth unless is reversed by a counter-mutation or another mutation that allows for cell growth
Mutation Darwinian or Adaptive? If Adaptive – Directed or Undirected?
C to T and G to A mutations via base tautomerism. Quantum mechanism?
Can DNA, and associated cell states, in the correct context (basis), be in a superposition of C/T, G/A?

## Slide 12
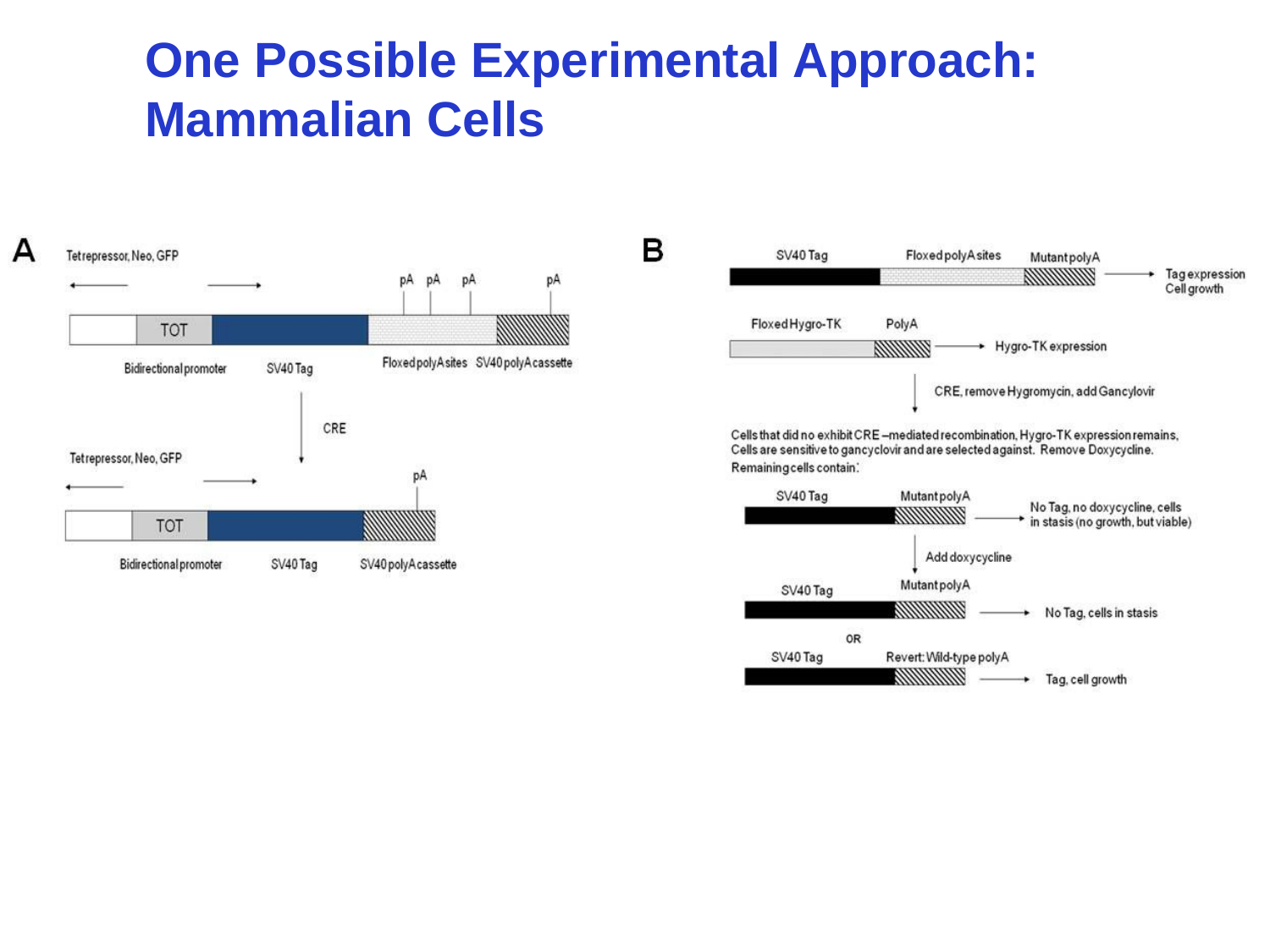

One Possible Experimental Approach:
Mammalian Cells

## Slide 13
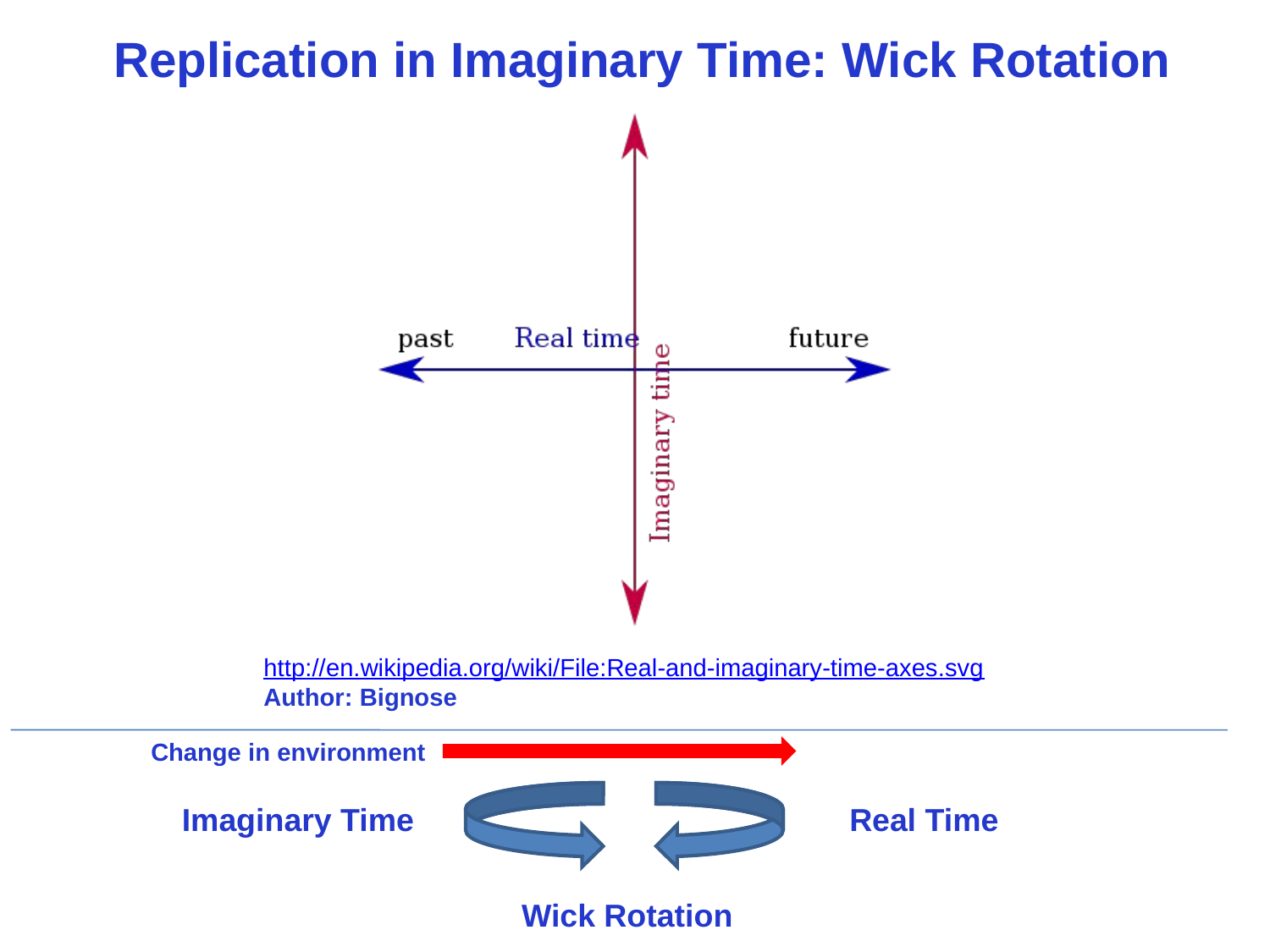

Replication in Imaginary Time: Wick Rotation
http://en.wikipedia.org/wiki/File:Real-and-imaginary-time-axes.svg
Author: Bignose
Change in environment
Imaginary Time
Real Time
Wick Rotation

## Slide 14
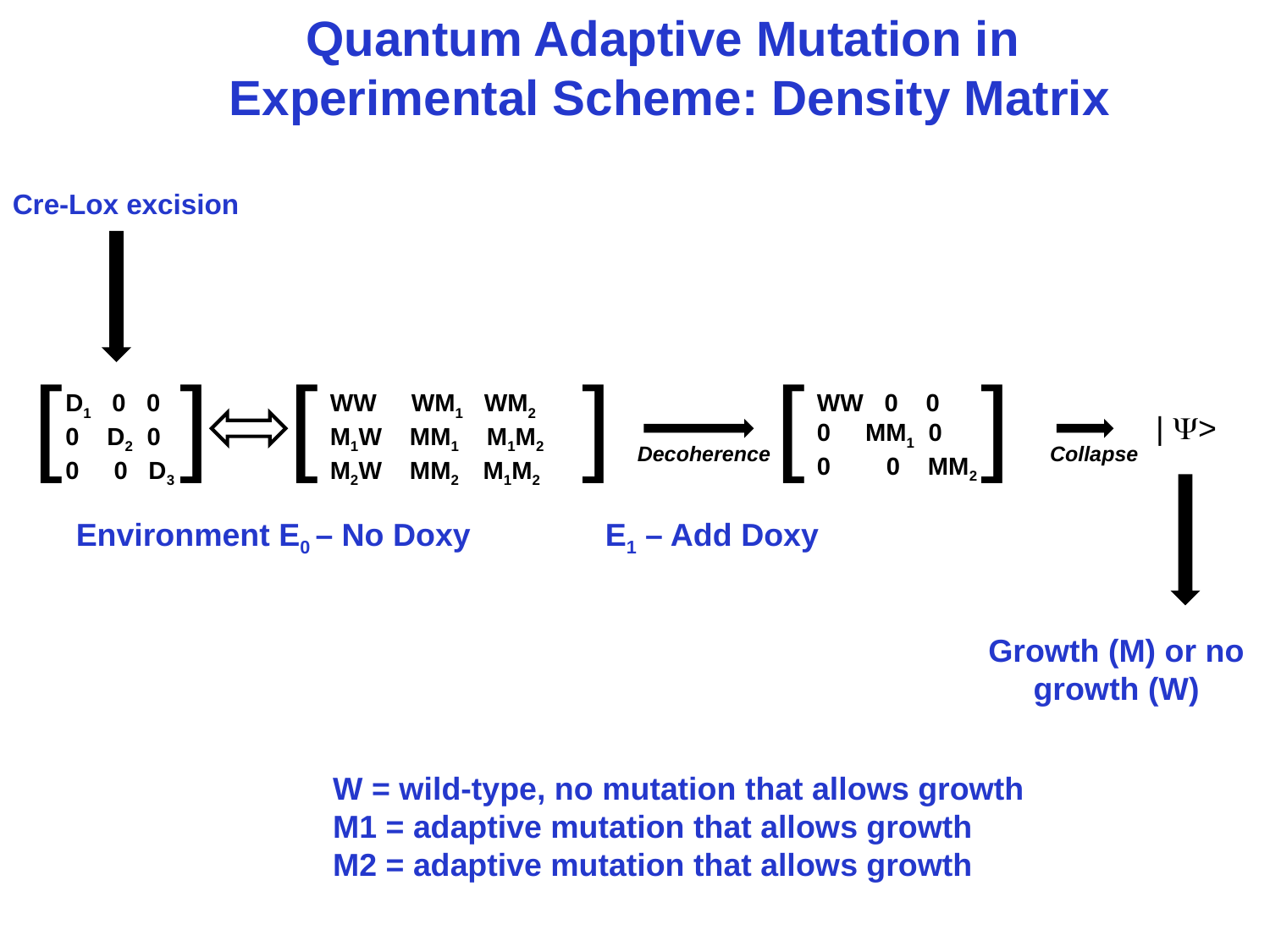

Quantum Adaptive Mutation in
Experimental Scheme: Density Matrix
Cre-Lox excision
[ ]
[ ]
[ ]
D1 0 0
0 D2 0
0 0 D3
WW WM1 WM2
M1W MM1 M1M2
M2W MM2 M1M2
WW 0 0
0 MM1 0
0 0 MM2
| >
Decoherence
Collapse
Environment E0 – No Doxy
E1 – Add Doxy
Growth (M) or no growth (W)
W = wild-type, no mutation that allows growth
M1 = adaptive mutation that allows growth
M2 = adaptive mutation that allows growth

## Slide 15
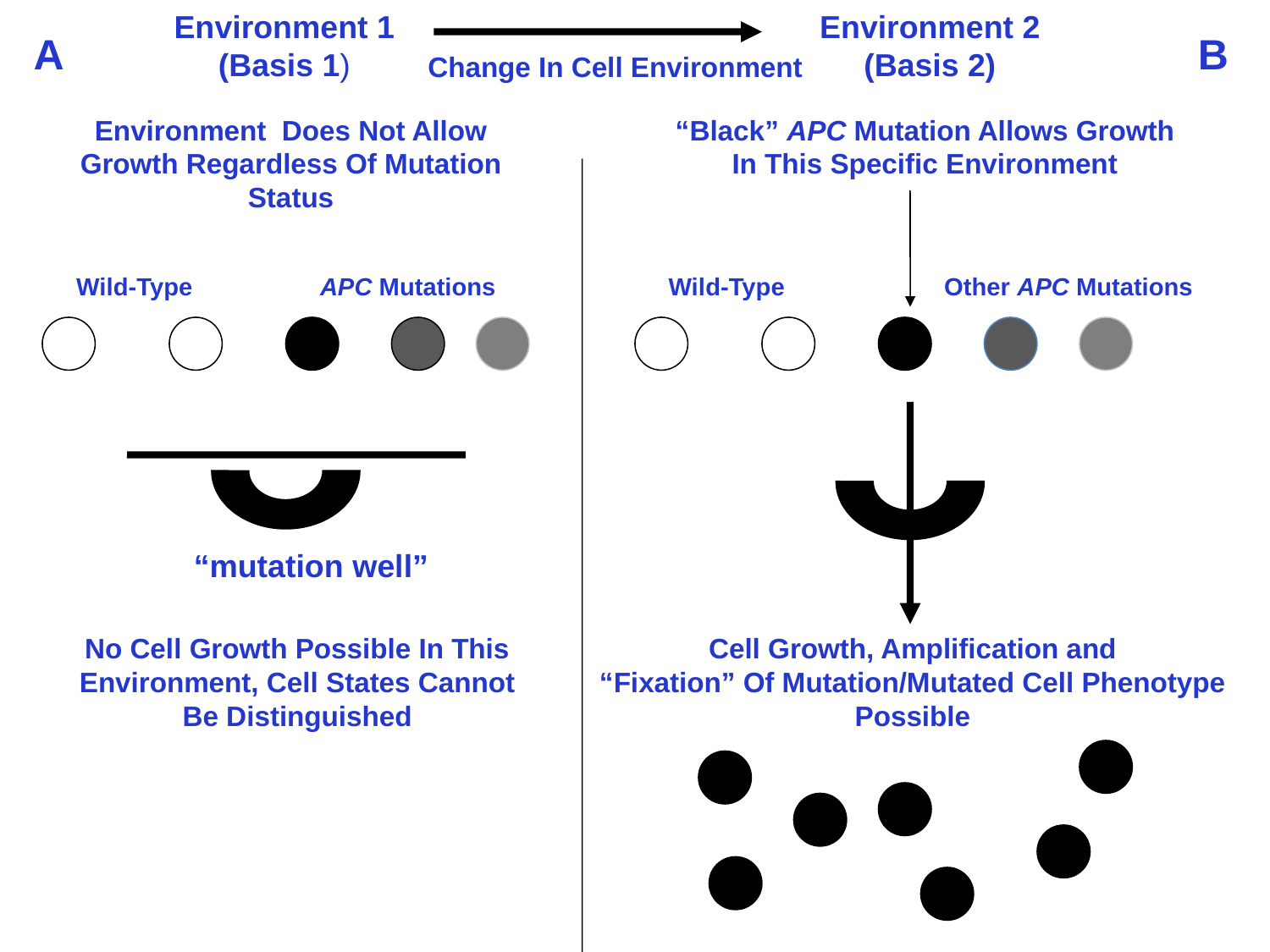

Environment 1
(Basis 1)
Environment 2
(Basis 2)
A
B
Change In Cell Environment
Environment Does Not Allow
Growth Regardless Of Mutation
Status
“Black” APC Mutation Allows Growth
In This Specific Environment
Wild-Type
APC Mutations
Wild-Type
Other APC Mutations
“mutation well”
No Cell Growth Possible In This
Environment, Cell States Cannot
Be Distinguished
Cell Growth, Amplification and
“Fixation” Of Mutation/Mutated Cell Phenotype
Possible

## Slide 16
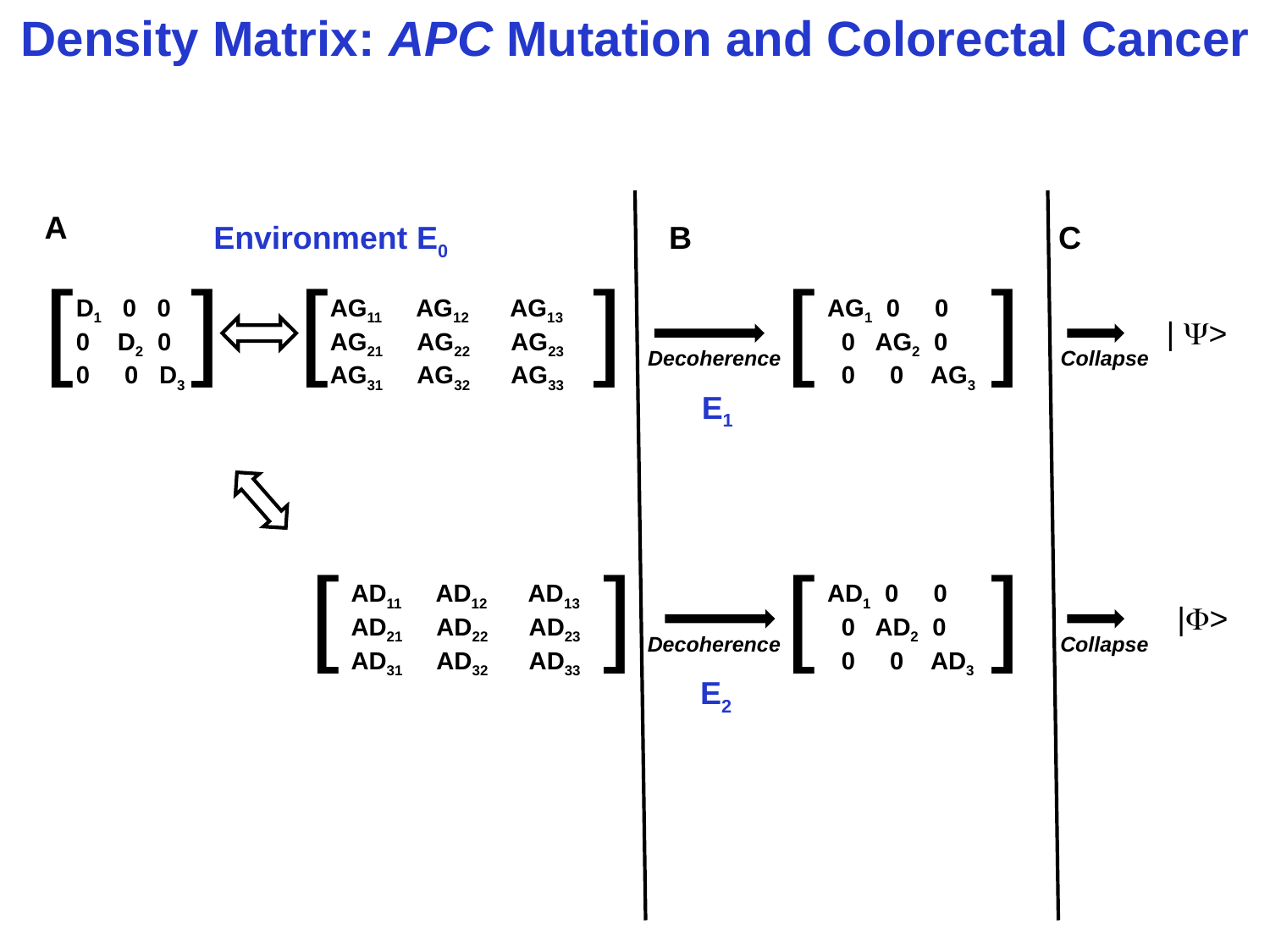

Density Matrix: APC Mutation and Colorectal Cancer
A
Environment E0
B
C
[ ]
[ ]
[ ]
D1 0 0
0 D2 0
0 0 D3
AG11 AG12 AG13
AG21 AG22 AG23
AG31 AG32 AG33
AG1 0 0
 0 AG2 0
 0 0 AG3
| >
Decoherence
Collapse
E1
[ ]
[ ]
AD11 AD12 AD13
AD21 AD22 AD23
AD31 AD32 AD33
AD1 0 0
 0 AD2 0
 0 0 AD3
|>
Decoherence
Collapse
E2
